# From Follicle Cell Differentiation and Structure to Chorion Biogenesis in Insects: Cellular Mechanisms, Gene Regulation, Biochemical Composition and Structural Diversity

**DOI:** 10.3390/insects17070659

**Published:** 2026-06-23

**Authors:** Thamara Rios, Isabela Ramos

**Affiliations:** 1 Instituto de Bioquímica Médica Leopoldo de Meis, Universidade Federal do Rio de Janeiro, Rio de Janeiro 21941-902, RJ, Brazil; thamara.rios@bioqmed.ufrj.br; 2 Instituto Nacional de Ciência e Tecnologia em Entomologia Molecular, Universidade Federal do Rio de Janeiro, Rio de Janeiro 21941-902, RJ, Brazil

**Keywords:** choriogenesis, insect chorion, insect eggshell, chorion biogenesis, follicle cells, chorion composition, eggshell ultrastructure

## Abstract

Choriogenesis, the final stage of oogenesis in insects, is a highly coordinated developmental process responsible for the formation of the chorion (eggshell), a specialized multilayered extracellular matrix that protects the embryo and mediates essential physiological functions. Despite its fundamental importance for reproductive success and species survival, the mechanisms underlying chorion biogenesis remain incompletely understood across insect taxa. This review provides an updated synthesis and integrated view of choriogenesis, including cellular, molecular, biochemical, and structural perspectives. We examine the role of follicle cells in chorion formation, the regulatory mechanisms governing chorion gene expression, and the biochemical composition of the eggshell, including proteins, lipids, and carbohydrates. In addition, we compare the structural diversity of the chorion across insect taxa, highlighting both conserved multilayered organization and lineage-specific adaptations in surface morphology and internal architecture.

## Simple Summary

Insects are among the most successful animals on Earth, occupying a wide range of environments and playing important roles in ecosystems, agriculture, and public health. Their reproductive success depends largely on the ability of their eggs to survive environmental challenges such as dehydration, temperature fluctuations, predators, and pathogens. This protection is provided by the eggshell, a specialized structure formed during the final stage of egg development. Although the insect eggshell has been studied for many decades, information about how it is produced remains scattered among different research fields and insect groups. In this review, we bring together current knowledge on the formation of the insect eggshell, from the cells that produce it and the genes that control its development to its chemical composition and structural organization. We also compare eggshell architecture among different insect groups, highlighting both shared features and unique adaptations associated with diverse lifestyles and habitats. By providing an integrated overview of eggshell formation, this review contributes to a better understanding of insect reproduction and evolution and may help identify new opportunities for the development of innovative and environmentally friendly strategies for the management of insect pests and disease vectors.

## 1. Introduction

Insects represent one of the most diverse and ecologically significant groups of organisms on Earth, playing fundamental roles in the functioning and stability of natural ecosystems. As pollinators, they facilitate the reproduction of a vast number of wild and cultivated plant species, supporting both biodiversity and agricultural productivity [1]. Predatory and parasitoid insects contribute to the natural regulation of animal populations [2], whereas detritivorous and necrophagous species accelerate the decomposition of organic matter and nutrient recycling [3]. Through these and many other ecological functions, insects sustain food webs, ecosystem productivity, and biogeochemical cycles, making them indispensable components of terrestrial and freshwater environments [4].

The remarkable diversity of insects is widely recognized as a key factor underlying their evolutionary success. This success is associated with several adaptive traits, including small body size, the exploitation of diverse food resources, high reproductive rates, and the ability to colonize a wide range of habitats [5]. However, some insect species also have profound impacts on human society. Many insect species act as vectors of pathogens affecting humans, animals, and plants, contributing significantly to the global burden of infectious diseases. Vector-borne diseases account for a substantial proportion of global infectious illnesses and are responsible for approximately 700,000 deaths annually, representing about 17% of the total burden of infectious diseases [6,7]. In addition to their role in disease transmission, insects also have major economic impacts as agricultural pests, causing extensive crop losses and reducing food production. It is estimated that insect pests are responsible for the loss of a significant fraction of global crop yields each year, both through direct plant damage and by transmitting plant pathogens [8]. Consequently, understanding insect biology and physiology is essential for developing strategies to mitigate their negative impacts and improve public health and agricultural productivity. Among the aspects of insect biology, the remarkable reproductive capacity largely depends on highly efficient mechanisms of egg production, which occur through a process known as oogenesis.

Oogenesis is the biological process responsible for the formation and maturation of oocytes (female gametes) within the female ovaries, ultimately resulting in the production of fertile eggs. This process is highly adaptive and is tightly regulated by nutritional and hormonal signaling cues [9,10]. In insects, oogenesis takes place within the ovarioles, which are the functional units of the ovaries. Each ovariole is divided into two main regions: the germarium, also referred to as the tropharium, an anterior region where oogonia (undifferentiated germline cells) divide and differentiate into oocytes; and the vitellarium, a posterior region where oocytes grow and accumulate nutrients that will be stored in the egg. Following the first meiotic division that leads to the differentiation of germ cells into an oocyte, oogenesis is initiated. Subsequently, the first major phase of oogenesis, known as vitellogenesis, begins. During this stage, there is large-scale synthesis and secretion of yolk protein precursors by the fat body and, to a lesser extent, by the ovaries. These precursors are released into the hemolymph and subsequently taken up by growing oocytes through receptor-mediated endocytosis. Once internalized, they are stored within specialized organelles known as yolk granules. The yolk is rich in reserve macromolecules, including proteins, lipids, and carbohydrates, which will later be mobilized during embryogenesis to support the nutritional demands of the developing embryo [9,11,12,13,14].

Following the completion of vitellogenesis, the developing oocyte enters the final stage of maturation known as choriogenesis. During this phase, the chorion (eggshell) is synthesized and secreted by the follicle cells, which form a secretory tissue of somatic origin that surrounds the oocyte during development. This single layer of follicle cells that envelops the oocytes is also known as the follicular epithelium (FE). Choriogenesis involves the coordinated synthesis, secretion, and assembly of numerous structural proteins and other macromolecules that give rise to the multiple layers of the chorion. This multilayered structure constitutes a highly specialized extracellular matrix that protects the developing embryo from environmental stresses such as desiccation, mechanical damage, and microbial invasion and also contributes to gas exchange and water balance during embryonic development [15,16,17,18,19]. Variations in chorion structure, thickness, ornamentation, and permeability reflect adaptations to different ecological niches and oviposition environments, such as aquatic habitats, arid terrestrial substrates, or host-associated microhabitats. Consequently, the remarkable diversity observed in insect eggshell architecture is considered an important evolutionary innovation that has contributed to the ecological success and diversification of insects across terrestrial ecosystems [20,21].

Despite decades of research, our understanding of many aspects of chorion biogenesis, including the mechanisms regulating protein synthesis, secretion, and extracellular assembly, remains fragmented. Here, we review current knowledge on the mechanisms underlying choriogenesis in insects, emphasizing chorion biosynthesis by follicle cells, the diversity of chorion composition and structure, and the genetic regulation of this process. We compile and compare available information from different insect groups in order to highlight both conserved mechanisms and lineage-specific adaptations involved in chorion formation. In addition, we discuss how recent advances in our understanding of choriogenesis, together with the availability of modern molecular tools, such as RNA interference (RNAi) and genome editing technologies like the CRISPR/Cas9 system, open new opportunities to investigate and manipulate the processes involved in eggshell formation and embryo viability. These approaches may ultimately contribute to the development of innovative strategies for controlling insect vectors of public health importance and agricultural pests.

## 2. General Structural and Functional Features of Follicle Cells in Insects

**Figure 1:**
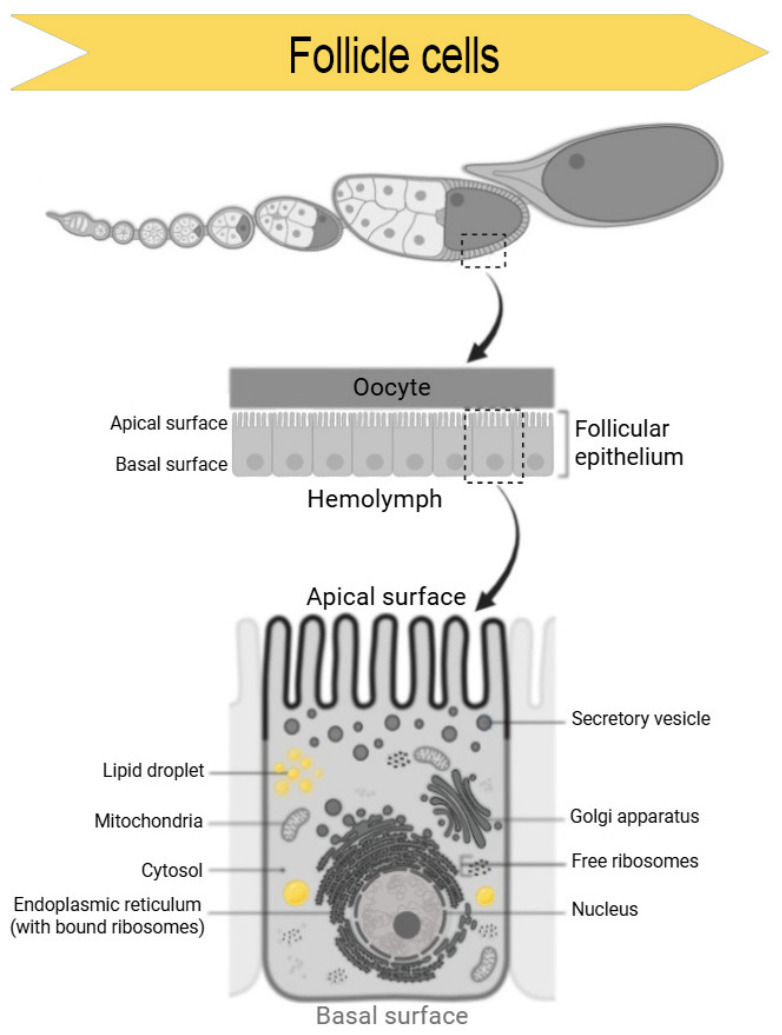
General organization and ultrastructural features of insect follicle cells. Schematic representation of the follicular epithelium surrounding the developing oocyte, highlighting the structural organization and secretory specialization of follicle cells. These cells form a polarized monolayered epithelium in which the apical surface faces the oocyte, while the basal surface is oriented toward the hemolymph. Ultrastructurally, follicle cells are characterized by a large nucleus with dispersed chromatin, abundant mitochondria, extensive rough endoplasmic reticulum, prominent Golgi complexes, and numerous secretory vesicles, reflecting their intense biosynthetic activity. The accumulation of secretory vesicles and the presence of apical membrane projections are associated with the synthesis, processing, and deposition of eggshell components during choriogenesis. The ovariole and egg chamber illustrations are based on the organization described for *D. melanogaster*.

Follicle cells constitute a highly polarized epithelial layer that surrounds the developing follicle and plays essential roles throughout insect oogenesis. Their structural and functional organization, more extensively characterized in *Drosophila melanogaster*, provides a general framework for understanding follicular epithelium biology across insect species. In general, ultrastructural analyses reveal that follicle cells possess large nuclei with dispersed chromatin, abundant mitochondria, extensive rough endoplasmic reticulum, and prominent Golgi complexes, consistent with their high biosynthetic and secretory activity. Secretory vesicles accumulate in the cytoplasm prior to and during chorion formation, highlighting their central role in eggshell biogenesis (Figure 1) [22].

The follicle cells also display a well-defined apical–basal polarity, with the apical surface facing the oocyte and often exhibiting microvilli or membrane protrusions that increase surface area for secretion and molecular exchange. In contrast, the basal domain interfaces with the basement membrane and hemolymph, enabling the uptake of nutrients and yolk precursors. Epithelial integrity is maintained by specialized junctional complexes, including septate junctions, adherens junctions, and gap junctions, which ensure tissue cohesion and controlled communication between cells. This organization supports the directional secretion of eggshell components and establishes a regulated interface between somatic follicle cells and the germline [22,23,24].

Beyond classical epithelial polarity, follicle cells also exhibit regional and functional specialization within the follicular epithelium. Studies in *D. melanogaster* have shown that distinct subpopulations of follicle cells arise along the anterior–posterior and dorsal–ventral axes of the egg chamber, acquiring specific morphologies and roles during development. This spatial patterning is accompanied by marked ultrastructural differentiation, particularly in the distribution of organelles involved in biosynthesis and secretion. The apical cytoplasm is typically enriched in secretory vesicles, while extensive rough endoplasmic reticulum and well-developed Golgi complexes reflect intense protein synthesis and trafficking activity. Additionally, dynamic remodeling of cytoplasmic architecture throughout oogenesis correlates with the temporal production and deposition of distinct eggshell layers [22,23,24,25].

Another common ultrastructural hallmark across multiple insect orders is the dynamic change in follicle cell morphology during oogenesis. During previtellogenesis, they are typically cuboidal and tightly connected through junctional complexes. As vitellogenesis proceeds and the oocyte increases in size, these cells often transition to a columnar morphology and later become squamous as they stretch to accommodate oocyte growth [26,27,28,29,30].

### 2.1. Follicle Cells Across Different Insect Groups

Although the primary roles of follicle cells in chorion synthesis are broadly conserved across insects, substantial variation exists in their morphology, differentiation, and additional functions among taxa, reflecting differences in ovariole structure, oogenesis, and ecological adaptations. Comparative studies reveal both conserved features and lineage-specific specializations, highlighting the evolutionary diversification of mechanisms underlying egg development and chorion formation [22].

**Figure 2:**
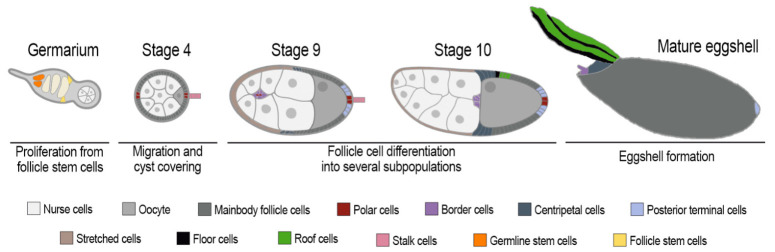
Sequential formation of the egg chamber and differentiation of follicle cell subpopulations in *D. melanogaster*. Schematic representation of the major developmental events involved in egg chamber formation and the establishment of specialized follicle cell populations. In the germarium, follicle stem cells give rise to proliferating follicle cells, which subsequently migrate and encapsulate the germline cyst composed of the oocyte and nurse cells, forming the egg chamber. As oogenesis progresses, follicle cells undergo spatially regulated differentiation into distinct subpopulations, including mainbody follicle cells, polar cells, border cells, centripetal cells, stretched cells, posterior terminal cells, roof cells, floor cells, and stalk cells. These cell types perform specialized morphogenetic and secretory functions during egg development, and collectively culminate in the formation of the mature eggshell.

Most of the current knowledge on follicle cell biology and eggshell formation in insects has been derived from studies conducted in holometabolous species. Among these, Diptera represents by far the most extensively investigated group, largely due to the availability of powerful genetic and molecular tools, in particular, *D. melanogaster* [30]. In this species, follicle cells form a monolayered somatic epithelium that surrounds the germline cyst composed of the oocyte and nurse cells, generating the egg chamber during oogenesis. The formation of the chamber involves three interdependent processes involving the cells of the follicular epithelium: (i) their proliferation from follicle stem cells located in the germarium; (ii) their migration and subsequent covering of a cyst formed by the developing oocyte and 15 nurse cells; and (iii) their differentiation into several specialized subpopulations with distinct morphogenetic and secretory roles. These subpopulations of follicle cells are: mainbody follicle cells (columnar follicle cells), polar cells, border cells, centripetal cells, stretched cells (squamous follicle cells), floor cells, roof cells and stalk cells [30,31,32]. The mainbody follicle cells constitute the majority of the epithelium and are responsible for the synthesis and deposition of most eggshell layers, including the vitelline membrane and chorion (which will be discussed later). At the anterior and posterior poles of the egg chamber, polar cells differentiate early and act as organizing centers that pattern surrounding follicle cells through signaling pathways such as Notch and JAK/STAT [31,33,34]. The anterior polar cells also induce the formation of border cells, a small migratory cell cluster that moves between nurse cells toward the oocyte during mid-oogenesis and contributes to eggshell patterning, such as the positioning of the micropyle, a structure for sperm entry [33,35]. Additional subpopulations include centripetal cells, which migrate inward between the oocyte and nurse cells to contribute to eggshell formation at the anterior region, stretched cells, which flatten over the degenerating nurse cells and participate in their removal during late oogenesis, and floor cells and roof cells that participate in the dorsal appendages formation, which are specialized structures that primarily facilitate gas exchange for the developing embryo [33,35]. The stalk cells organize into a short linear column positioned between adjacent egg chambers within each ovariole. The primary function of stalk cells is to physically separate successive developing follicles, thereby maintaining the characteristic linear arrangement of egg chambers along the ovariole (Figure 2) [31,33,34]. Transmission electron microscopy studies have shown that follicle cells exhibit typical characteristics of metabolically active secretory epithelial cells. Their single nucleus is usually large and irregular, containing dispersed chromatin material. Besides, the cytoplasm is rich in mitochondria, concentric lamellae of rough endoplasmic reticulum surrounding lipid droplets and well-developed Golgi complexes. Numerous secretory vesicles accumulate in the cytoplasm, particularly during late oogenesis, when eggshell components are synthesized and transported to the apical plasma membrane facing the oocyte [36,37,38].

Even within higher dipterans (Brachycera), including the model *D. melanogaster*, follicle cell differentiation can vary significantly, indicating that mechanisms of follicular epithelium patterning and morphogenesis are highly diversified, even among closely related species. In more derived brachycerans, including *D. melanogaster*, several follicle cell populations, such as border cells, centripetal cells, and mainbody follicle cells, exhibit migratory behavior during oogenesis. In contrast, in most representatives of the infraorder Orthorrhapha (lower brachycerans), the only follicle cells that retain migratory capacity are the border cells, which actively migrate from the anterior region of the follicle between the nurse cells toward the anterior pole of the oocyte. This restricted migratory behavior results in the absence of certain follicle cell types and, consequently, the lack of specific structural specializations in the eggshell [30,39].

In the so-called lower dipterans (suborder Nematocera), clear differences arise in the degree of cellular diversification. In representatives of the infraorders Tipulomorpha and Trichoceromorpha, only a limited number of follicle cell types have been described, with four main populations recognized: anterior and posterior polar cells, anterior terminal cells, and mainbody follicle cells. This reduced diversification appears to be associated with the limited capacity for cell migration in these taxa. Rather than undergoing active migration, follicle cells can only modify their relative positions within the epithelium through changes in cell shape and consequently do not invade the compartment containing the nurse cells, a process characteristic of follicle cell dynamics in *D. melanogaster* [40]. A representative example of this pattern can be observed in mosquitoes belonging to the family Culicidae. In these insects, the follicular epithelium is monostratified and surrounds both the oocyte and the nurse cells. In *Aedes aegypti*, one of the most extensively studied members of this family, follicle cells exhibit the differentiation pattern typical of nematoceran dipterans [40]. Interestingly, during the previtellogenic stage, these cells are characterized by a large nucleus and a cytoplasm relatively poor in organelles. A few hours after blood feeding, however, follicle cells undergo marked ultrastructural changes and display cytoplasm rich in free ribosomes, mitochondria, an extensively developed rough endoplasmic reticulum, and prominent Golgi complexes containing vesicles filled with electron-dense material [41,42,43,44]. When compared with the complex pattern of follicle cell diversification described in *D. melanogaster* and other representatives of the suborder Brachycera, the follicular epithelium of nematoceran species such as mosquitoes appears relatively simple. In particular, the extensive migration events and the large diversity of follicle cell subpopulations characteristic of higher dipterans are largely absent [40,42,44].

Another group of holometabolous insects that has been extensively investigated comprises species of the order Lepidoptera. In these insects, the follicular epithelium originates as a monolayer of flattened cells covering the nurse cells and cuboidal or columnar cells surrounding the oocyte, whose morphology changes throughout successive stages of follicular development [29]. In the flour moth *Ephestia kuehniella*, chorion components begin to be deposited by follicle cells during developmental stage 6, when the cytoplasm of these cells becomes filled with membrane-bound electron-dense granules, ribosomes, and mitochondria [45,46]. The first detailed description of follicular epithelium differentiation and diversification in butterflies was reported for *Pieris napi*. In the polytrophic ovaries of this species (characterized by germ cells that differentiate into both oocytes and specialized nurse cells, with the nurse cells traveling alongside the developing oocyte and remaining directly connected to it, such that each follicle contains one oocyte and its own cohort of nurse cells) [47], follicle cells differentiate into five subpopulations: mainbody follicle cells, stretched cells, posterior terminal cells, centripetal cells, and interfollicular stalk cells. Among these, the centripetal cells display migratory behavior and are responsible for the formation of the micropyle. In addition, the cytoplasm of follicle cells, particularly those of the mainbody, is enriched with numerous mitochondria, cisternae of the endoplasmic reticulum, Golgi complexes, and many vesicles containing electron-dense material [47].

In the most extensively studied lepidopteran species, the silkworm *Bombyx mori*, differentiation of the follicle cells begins in the basal region of the germarium and initially leads to the formation of two follicle cell subpopulations: columnar cells covering the oocyte and cuboidal cells surrounding the nurse cells. Follicle cells located near the nurse-cell compartment subsequently acquire migratory capacity and give rise to a subpopulation of centripetal cells that migrate between the two compartments to form the micropyle. Thus, three main follicle cell subpopulations are generally recognized in this species: cells covering the oocyte, cells surrounding the nurse cells, and centripetal cells that migrate between these compartments [48]. An interesting specialization occurs within the centripetal cell population. The micropylar apparatus is formed by three specialized types of centripetal follicle cells: the micropylar channel-forming cells (MCFCs), the micropylar orifice-forming cells (MOFCs), and the micropylar rosette-forming cells (MRFCs) [49]. Finally, during stage 11 of follicular development, when the process of choriogenesis begins, the follicular epithelium displays a cytoplasm rich in well-developed rough endoplasmic reticulum, Golgi complexes, and secretory vesicles, a pattern that persists until the completion of chorion production at stage 12 [48].

Compared to holometabolous insects, follicle cell organization and function in hemimetabolous species remain poorly understood, representing a key gap given their ecological and medical importance, including hemipteran vectors like *Rhodnius prolixus*. In the telotrophic meroistic ovary of this species (unlike polytrophic meroistic ovaries, nurse cells remain in the tropharium and are connected to developing oocytes via nutritive cords [22]), follicle cells originate from somatic stem cells located in the tropharium. In the prefollicular epithelium of nymphs, these cells appear flattened and contain regularly distributed organelles, as well as lipid droplets and glycogen reserves [18]. In the adult insect, these cells initially form a syncytial prefollicular epithelium, which subsequently gives rise to a layer of columnar follicle cells surrounding previtellogenic oocytes. By the end of previtellogenesis, follicle cells become binucleated. During vitellogenesis, follicle cells retain a binucleated columnar morphology and differentiate into two morphologically distinct regions: the cap or apical end cells, which form a thicker epithelium composed of closely apposed cells, and lateral follicle cells, which cover the oocyte surface and form a thinner epithelium with more widely spaced and apparently shorter cells [18]. During choriogenesis, follicle cells remain binucleated but adopt a cuboidal shape and become tightly apposed once again, with reduced intercellular spaces [18]. Throughout oogenesis, follicle cells exhibit abundant mitochondria, well-developed Golgi complexes, numerous ribosomes, and extensive rough endoplasmic reticulum, reflecting intense biosynthetic and secretory activity consistent with the ultrastructural features of professional secretory cells [18]. However, notable cytoplasmic changes occur during the transition from vitellogenesis to choriogenesis. During vitellogenesis, follicle cells display enlarged endoplasmic reticulum tubules with an atypical vesicular morphology, as well as smaller and more elongated mitochondria. In contrast, during choriogenesis, mitochondria become larger and more rounded, while endoplasmic reticulum cisternae are reduced in size (approximately 80% smaller), although still exhibiting a vesicular organization and occupying a larger proportion of the cytoplasm [50]. Additionally, follicle cells contain clusters of small electron-dense granules, which are presumed to represent precursors of chorion components [18].

In *Mahanarva fimbriolata*, another insect belonging to the order Hemiptera, follicle cells also exhibit changes in intercellular spacing throughout oogenesis, with more advanced stages of vitellogenesis characterized by enlarged intercellular spaces within the follicular epithelium. In addition, these cells display a cytoplasm rich in mitochondria and two distinct forms of rough endoplasmic reticulum: the classical lamellar type and, similarly to what has been described in *R. prolixus*, a more atypical vesicular form. The coexistence of these two types of rough endoplasmic reticulum within the same cells may indicate functional differentiation, with the vesicular form being interpreted as a more active configuration, more directly associated with protein synthesis when compared to the lamellar form, although both are likely involved in this process [51].

Additional examples of hemimetabolous insects exhibiting the follicular epithelium characteristics described above can be found within the order Orthoptera. In the desert locust *Schistocerca gregaria*, follicle cells during vitellogenesis display a cytoplasm containing relatively low amounts of rough endoplasmic reticulum and small Golgi complexes with cisternae lacking electron-dense material, but abundant free ribosomes and mitochondria. At the end of vitellogenesis, concomitant with the initial stages of eggshell formation, follicle cells undergo marked morphological changes, including a reduction in cell height and an increase in cell width. These changes are accompanied by cytoplasmic remodeling, characterized by an increase in the number of rough endoplasmic reticulum cisternae, the occasional presence of electron-dense material within Golgi cisternae, and a predominance of electron-dense vesicles in the cytoplasm. During the final stages of choriogenesis, a reduction in the amount of endoplasmic reticulum is observed, indicating a shift in cellular activity as eggshell formation progresses [52]. Something similar occurs in the house cricket *Acheta domesticus*. Throughout vitellogenesis, the follicular epithelium progresses from a columnar to a cuboidal shape and finally to squamous. The cytoplasm is filled with lipid droplets, mitochondria, ribosomes, Golgi cisternae, and rough endoplasmic reticulum. At the end of vitellogenesis, already with a squamous shape, there is an increase in the frequency of vesicular and multivesicular bodies in the cell cytoplasm, probably a preparatory moment for the next highly secretory stage of choriogenesis [23].

Table S1 compiles representative studies from different insect groups, which consistently report the presence of well-developed secretory machinery, such as rough endoplasmic reticulum, Golgi complex, and electron-dense vesicles, associated with the synthesis and export of chorion components. These findings reinforce the conserved function of follicle cells as the primary source of eggshell material across diverse insect taxa, despite variations in ovarian organization and follicular architecture.

### 2.2. Additional Functions of the Follicle Cells

Traditionally, follicle cells have been described as somatic components of the ovarian follicle responsible for synthesizing the eggshell. However, accumulating studies have revealed that these cells can also perform a number of additional functions during oogenesis.

One of the additional functions of follicle cells, and probably the most extensively studied besides their role in chorion synthesis, is their participation in supporting oocyte development by allowing the uptake of yolk components. The growth of the developing oocyte occurs through the uptake and storage of yolk protein precursors (YPPs) in yolk granules via receptor-mediated endocytosis at the oocyte surface. For this process to occur, YPPs circulating in the hemolymph must first reach the oocyte surface. This is made possible by changes in the patency of the follicular epithelium, which regulates its permeability. During vitellogenesis, the follicular epithelium becomes patent, allowing the paracellular transport of YPPs through enlarged intercellular spaces between follicle cells until they reach the receptors located on the oocyte surface. Studies have shown that the intercellular spaces within the follicular epithelium increase during the vitellogenic phase and subsequently decrease at the end of vitellogenesis and the onset of choriogenesis, when the epithelium regains its barrier properties [13,53,54,55,56,57,58].

In addition to paracellular transport, a transcellular route, occurring through the follicle cells themselves via transcytosis, has also been reported in certain social species of the order Hymenoptera [59,60] as well as in species belonging to the order Hemiptera [61]. These observations suggest that, in some taxa, YPP may traverse the follicular epithelium not only through intercellular pathways but also by being internalized and transported across the cytoplasm of follicle cells before reaching the oocyte surface. Furthermore, beyond the simple passage of yolk components through the follicular epithelium, either via paracellular transport or transcytosis, there is evidence suggesting that follicle cells themselves may contribute to synthesizing and secreting yolk components. Although the fat body is generally considered the primary site of YPP production in insects [11,14,62,63,64,65], several studies indicate that the ovary, and specifically the follicular epithelium, may also participate in this process in certain species. Evidence supporting follicle cell-derived vitellogenin has been reported in insects such as *R. prolixus* [66], *D. melanogaster* [67,68], and *Culex quinquefasciatus* [69].

In some taxa, the follicular epithelium exhibits specific specializations; for example, follicle cells may partially substitute for the chorion as a protective coating. In the gall midge *Heteropeza pygmaea*, which can reproduce through paedogenesis (larval parthenogenesis), oogenesis proceeds without chorion formation, and the embryos remain encapsulated by a persistent follicular epithelium. Follicle cells are interconnected by expanding systems of desmosomes and septate junctions aligned with the egg’s long axis, and display large nucleoli, abundant ribosomes, and cytoplasmic reserves of lipid droplets and glycogen, consistent with both mechanical and metabolic support roles rather than extensive extracellular matrix secretion [70].

Another example is the viviparous insect *Hemimerus talpoides*, in which follicle cells exhibit a placenta-like function. In this species, follicle cells do not degenerate after the complete maturation of the oocyte and the onset of embryogenesis. Instead, they undergo morphological modifications and form the wall of the chamber in which the embryo develops, remaining in direct contact with the embryonic tissues [71]. In *D. melanogaster*, a subset of follicle cells functions as non-professional phagocytes, being responsible for the clearance of degenerating nurse cells during the final stages of oogenesis. This process involves molecular pathways typically associated with phagocytosis, including receptors responsible for the recognition of apoptotic cells and the activation of cytoskeletal remodeling mechanisms that enable the engulfment and degradation of cellular remnants [72,73,74,75,76].

## 3. Regulation of Chorion Gene Expression

Building upon the ultrastructural and functional features of follicle cells involved in chorion biosynthesis, it becomes essential to consider the regulatory mechanisms that control the expression of chorion genes. The production of eggshell components is not solely dependent on the secretory capacity of follicle cells, but also on the precise temporal and spatial regulation of gene expression within this epithelium.

It is known that chorion genes are activated in a highly coordinated manner during the late stages of oogenesis, particularly at the onset of choriogenesis, and their expression is tightly restricted to specific subpopulations of follicle cells. This regulation is achieved through the integration of multiple levels of control, including hormonal signaling, especially ecdysone-dependent pathways, and the action of specific transcription factors interacting with conserved cis-regulatory elements [77,78,79]. In addition, signaling pathways that govern follicle cell differentiation and patterning, such as EGFR, Notch, and JAK/STAT, contribute to the spatial refinement of chorion gene expression [31,33,34]. Importantly, most of the current knowledge on the regulation of chorion gene expression derives from studies conducted in model holometabolous insects, particularly *D. melanogaster* and *B. mori*, in which the genetic and molecular bases of oogenesis have been extensively characterized. These studies have demonstrated that chorion gene expression is controlled by a multilayered regulatory network that integrates developmental timing, positional information within the follicular epithelium, and gene-specific regulatory architecture [77]. In this context, the regulation of chorion gene expression can be understood as the result of the coordinated action of several interconnected mechanisms, including developmental stage-dependent (temporal) programming, spatial regulation associated with cell identity and position, the activity of cis-regulatory elements, the combinatorial action of transcription factors, gene amplification, and the integration of hormonal and cell signaling pathways [77]. The following sections address each of these regulatory levels in detail.

### 3.1. Temporal and Spatial Regulation of Chorion Gene Expression

The expression of chorion genes in insects is tightly regulated in both temporal and spatial dimensions, ensuring the precise coordination between follicle cell differentiation and eggshell formation. In well-established model organisms, such as *D. melanogaster* and *B. mori*, chorion gene expression is mostly activated during the late stages of oogenesis [77].

**Figure 3:**
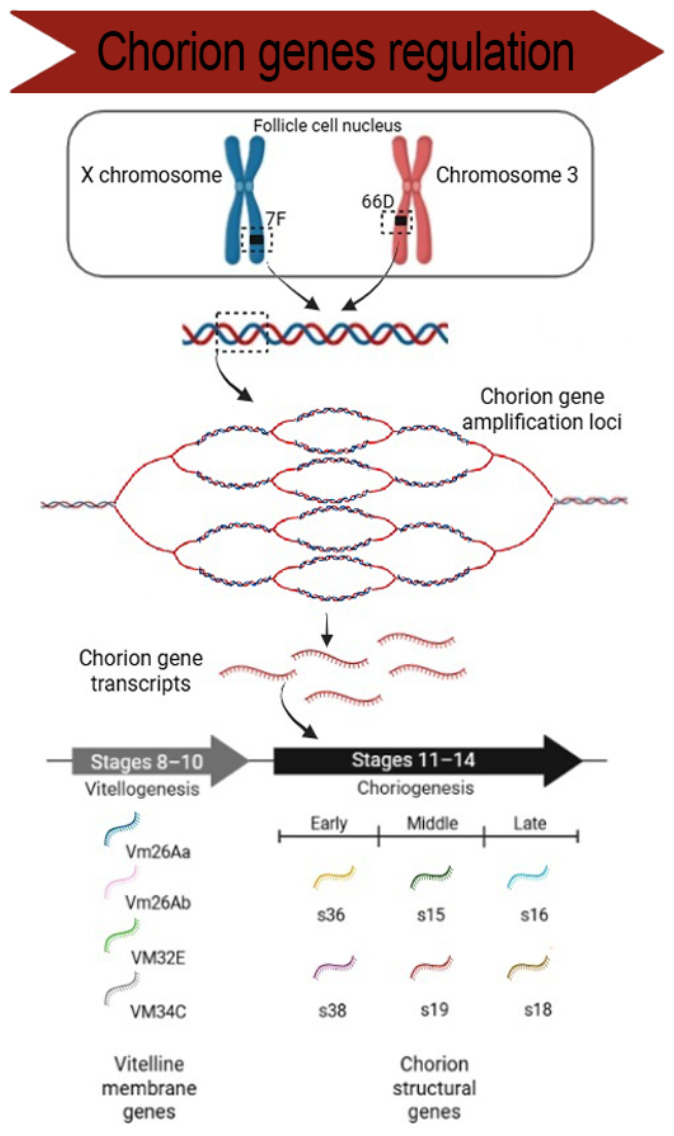
Temporal regulation and amplification of chorion gene expression during eggshell formation in *D. melanogaster*. Schematic representation of the coordinated transcriptional program underlying vitelline membrane and chorion formation during late oogenesis. During vitellogenesis (stages 8–10), follicle cells express vitelline membrane genes, which encode the proteins constituting the first eggshell layer deposited around the oocyte. As oogenesis progresses into choriogenesis (stages 11–14), chorion gene loci located in region 7F of the X chromosome and region 66D of chromosome 3 undergo locus-specific amplification, increasing gene dosage and supporting the exceptionally high transcriptional output required for rapid eggshell synthesis. Concomitantly, chorion structural genes are activated in a temporally ordered manner and can be classified into early, middle, and late expression groups. The sequential activation of these gene sets ensures the proper timing of chorion protein production and the orderly assembly of the mature eggshell.

In *D. melanogaster*, chorion genes are expressed in a stage-specific manner, with distinct subsets of genes being sequentially activated during late oogenesis. The proteins that constitute the vitelline membrane, a thin and relatively uniform non-cellular proteinaceous layer that constitutes the first eggshell layer deposited around the oocyte, are transcribed during vitellogenesis (developmental stages 8–10). This eggshell layer is mainly composed of a small set of structurally related proteins, including sV17 (Vm26Aa), sV23 (Vm26Ab), VM32E, and VM34C, which share a conserved domain in the vitelline membrane [77,79,80,81,82]. The amplification of chorion genes is initiated during the early stages of choriogenesis and persists throughout its progression, spanning approximately stages 11 to 14 of oogenesis. The genes encoding the major chorion proteins are organized into two distinct genomic clusters: one located at region 7F on the X chromosome and the other at region 66D on the third chromosome. These genes are temporally classified based on their expression profiles into early (*s36*, *s38*), middle (*s15*, *s19*), and late (*s16*, *s18*) chorion genes, reflecting their sequential activation during choriogenesis (Figure 3). Furthermore, similar amplification is observed at chromosomal loci 30B and 62D, appearing to contain additional genes for chorion formation [77,79,83,84,85,86]. Spatial regulation further refines chorion gene expression within the follicular epithelium. Follicle cells are organized along the anterior–posterior and dorsal–ventral axes of the egg chamber, and distinct subpopulations exhibit specific gene expression profiles that contribute to regional specialization of the eggshell. For example, genes involved in the formation of dorsal appendages and the micropyle are expressed in spatially restricted domains, reflecting the integration of positional information mediated by signaling pathways such as EGFR, Notch, and JAK/STAT. These pathways regulate follicle cell fate and patterning, thereby controlling the localized expression of chorion genes [31,32,34,79,87,88].

In *B. mori*, information regarding vitelline membrane genes remains relatively limited. However, similarly to what has been described in *D. melanogaster*, these genes are expressed prior to the onset of choriogenesis. Four major vitelline membrane genes have been identified within a syntenic genomic region, namely *BmEP80*, *BmVMP25*, *BmVMP23*, and *BmVMP30* [77,89]. In contrast, chorion genes in *B. mori* are considerably better characterized. These genes are located within a single continuous locus and are organized into pairs encoding two distinct branches (A and B), rather than being arranged in tandem as observed in *D. melanogaster*. Chorion genes in this species exhibit coordinated expression and can be classified into three main gene families within each branch: ErA/ErB, A/B, and HcA/HcB. The A branch comprises the A, ErA, and HcA gene families, whereas the B branch includes the B, ErB, and HcB families. The expression of these gene families follows a strict temporal sequence during choriogenesis. Proteins encoded by the ErA/ErB genes are synthesized during early choriogenesis (stages +1 to +8 of a typical ovariole containing a chorion-forming segment of approximately 40 follicles), whereas proteins from the A/B family are produced during middle choriogenesis (stages +6 to +28). In turn, HcA/HcB proteins are synthesized during late choriogenesis (stages +26 to +35), with follicles being considered mature after stage +36. Most chorion genes are arranged as A- and B- type gene pairs that share similar developmental specificity and are divergently transcribed from a common promoter region, resulting in coordinated gene expression within each pair [77,90,91,92,93]. However, a small subset of genes does not follow this paired organization. Instead, these genes occur as single transcriptional units interspersed between gene pairs, forming a distinct subfamily with independent regulatory characteristics [94].

### 3.2. Gene Amplification for Transcript Production—Drosophila as a Model

The precise temporal and spatial regulation of chorion gene expression ensures that eggshell components are synthesized in the appropriate cell populations and at the correct stages of oogenesis. In *Drosophila*, the exceptionally high demand for chorion proteins within a relatively short developmental window has driven the evolution of a specialized regulatory strategy in which gene activation is coupled to processes that enhance transcriptional capacity. In this context, gene amplification emerges as a key strategy to increase transcriptional output in follicle cells, linking developmental regulation with the quantitative requirements of chorion biosynthesis. Overall, the temporal and spatial patterns of chorion gene expression are closely associated with the amplification of multiple copies of chorion gene loci prior to transcription. This process enables a rapid increase in transcriptional output, thereby compensating for the limited time available for protein synthesis required for eggshell formation. During this period, follicle cells undergo polyploidization, beginning around stage 7 of egg chamber development and continuing endoreplicative cycles until approximately stage 11. However, interestingly, gene amplification does not occur uniformly across the genome. Loci located on the X chromosome are typically amplified approximately 16-fold, whereas those on the third chromosome can reach amplification levels of up to ~60-fold [79,83,85].

Importantly, chorion gene amplification should not be considered a universal feature of insect choriogenesis. Rather, it represents a highly derived mechanism that has been extensively characterized in *Drosophila* and related dipterans. In contrast, other insect groups have evolved alternative strategies to achieve the high levels of chorion gene expression required for eggshell formation. For example, in *B. mori*, more than 100 chorion genes are organized within a large continuous genetic locus on chromosome 2, and elevated chorion protein production is achieved through the coordinated expression of this multigene family rather than through extensive gene amplification. Thus, the chorion genes of dipterans are amplified to reach high levels of expression, while *B. mori* relies on the expression of a large repertoire of pre-existing chorion gene copies encoded in its genome [93,95].

### 3.3. Transcriptional Regulatory Architecture of Chorion Genes

The transcriptional activity of chorion genes is governed by a complex regulatory architecture involving the interaction of multiple molecular components. Among the regulatory components, cis-regulatory elements are fundamental in establishing the genomic context that enables transcription factors and signaling pathways to exert precise spatiotemporal control over gene expression. These elements, including promoters and enhancers, are generally located in promoter regions and consist of conserved sequence motifs that serve as binding sites for regulatory proteins, thereby integrating developmental and hormonal signals. In *D. melanogaster*, multiple cis-acting elements have been characterized within chorion gene loci, including the 320 bp Amplification Control Element on the third chromosome (ACE3), four Amplification Enhancing Regions (AERs) and replication origins such as ori-α, ori-β, and ori-γ. Together, these elements regulate locus-specific gene amplification, thereby increasing gene dosage and supporting the exceptionally high levels of chorion gene transcription required during eggshell formation [79,83,96,97,98,99,100,101]. Studies in *B. mori* have also demonstrated the presence of cis-regulatory motifs within short bidirectional promoters associated with divergently transcribed A–B gene pairs, which contribute to the coordinated expression of paired genes during distinct phases of choriogenesis. Notably, many of these cis-regulatory elements appear to be functionally conserved between *D. melanogaster* and *B. mori*, despite differences in genomic organization and regulatory context [90,93,102,103,104,105]. Collectively, these findings illustrate how cis-regulatory elements integrate transcriptional control with promoter architecture, thereby ensuring the tightly regulated production of chorion components.

In *D. melanogaster*, key trans-regulatory elements include members of the Broad-Complex (BR-C), which act downstream of hormonal cues and are essential for stage-specific gene expression [106,107], as well as factors such as CF1 and CF2 [83,108] and Tramtrack69 (Ttk69) [109]. In addition, CCAAT/enhancer-binding proteins (C/EBPs) act as trans-regulatory elements capable of binding chorion gene promoters and are also involved in border cell migration during oogenesis [77,110,111]. In *B. mori*, similar regulatory principles are observed, with C/EBP-like factors playing central roles in the activation of chorion gene promoters. In addition, members of the GATA family of transcription factors have been identified as key trans-regulatory elements involved in this process, particularly within bidirectional promoter regions associated with divergently transcribed A–B gene pairs [95,102,112,113,114,115]. Furthermore, chromatin-associated factors such as HMGA proteins and the chromatin remodeler CHD1 also act as trans-regulatory elements by modulating chromatin architecture and facilitating the recruitment of transcriptional machinery [104,116].

Importantly, as discussed above, chorion gene expression is organized into temporally distinct waves, with specific subsets of early, middle, and late genes being sequentially activated during choriogenesis in both *D. melanogaster* and *B. mori*. Consequently, the transcription factors that regulate these genes can also exhibit temporal specificity, ensuring that each group of chorion genes is expressed at the appropriate developmental stage. In *B. mori*, C/EBP acts as a major regulator of early/early-middle chorion genes. Interestingly, because it recognizes both early- and late-type binding sites, C/EBP also functions as a repressor of late chorion genes during the initial stages of choriogenesis, thereby preventing their premature activation and serving both activating and repressive functions within the chorion gene regulatory network. In contrast, GATA transcription factors are closely associated with the expression of late chorion genes and are activated concomitantly with the Hc chorion gene families [77,95,102,112,113]. Additional regulatory factors, including the proteins HMGA and CHD1, contribute to the temporal control of chorion gene expression by modulating chromatin architecture and inducing structural changes in promoter regions associated with middle chorion genes [77,104,116]. Similar temporal regulation has also been documented in *D. melanogaster*. For example, the transcription factors CF1 and CF2 bind to the promoter region of the middle chorion gene *s15*, contributing to its stage-specific expression [108]. Furthermore, C/EBP is required for border cell migration during stages 9–10 of oogenesis. Upon reaching the anterior pole of the oocyte, these cells participate in micropyle formation, indicating that C/EBP contributes to the regulation of developmental processes associated with choriogenesis even before the onset of chorion deposition itself [110,111].

A central upstream regulator of this network is hormonal signaling mediated by ecdysone (20-hydroxyecdysone), which plays a pivotal role in coordinating the transition from vitellogenesis to choriogenesis. Beyond its role in transcriptional activation, ecdysone signaling is also critically involved in the morphogenesis of the follicular epithelium, regulating changes in cell shape, polarity, and differentiation, as well as orchestrating the migration of specific follicle cell subpopulations required for proper egg chamber patterning. Through the activation of a hierarchical cascade of downstream transcription factors, including members of the BR-C and other early-response genes, ecdysone integrates systemic endocrine cues with local cellular behaviors, thereby coupling epithelial remodeling and cell migration to the precise activation of chorion structural genes [77,106,117,118,119,120]. However, the contribution of hormonal signaling to choriogenesis is not conserved across insect taxa and displays important lineage-specific differences. While elevated ecdysone signaling promotes key events during late oogenesis and choriogenesis in Diptera, particularly in *D. melanogaster*, alternative regulatory patterns have been reported in other insect groups. In *B. mori*, for example, the onset of choriogenesis is associated with a decline in ecdysone signaling [77,121,122]. A similar pattern has been observed in *A. aegypti*, where the progression of choriogenesis is accompanied by reduced expression of several components of the ecdysone signaling pathway, including ecdysone receptor (*EcR*), Ultraspiracle (*USP*), ecdysone-induced protein 74EF isoform B (*E74B*), ecdysone-inducible gene E75, and the early-late ecdysone-inducible gene *HR3* [123]. In contrast, ecdysteroid signaling is essential for proper chorion formation in *D. melanogaster*, where components of the ecdysone cascade, such as EcR and USP, regulate processes including border cell migration and chorion gene amplification through interactions with regulatory elements of chorion loci [77,79,118]. Similar functions have been reported in the beetle *Tribolium castaneum*, in which EcR and USP contribute to follicle cell growth and migration during oogenesis [124]. Likewise, in *R. prolixus*, silencing of these genes resulted in a marked reduction in the expression of Rp30 and Rp45, the two major chorion proteins of this species, leading to the production of collapsed eggs with reduced hatching rates and highlighting the importance of ecdysone signaling for proper chorion biogenesis [125].

### 3.4. Evolution and Functional Diversification of Chorion Genes

Beyond their developmental importance, chorion genes provide a compelling example of the interplay between functional conservation and evolutionary diversification. Although the primary role of the eggshell in protecting the developing embryo is broadly conserved across insects, chorion genes frequently exhibit rapid evolutionary rates, lineage-specific expansions, and substantial variation in gene family organization. This diversification is thought to be driven by selective pressures associated with distinct reproductive environments and oviposition strategies, including adaptation to desiccation, aquatic habitats, mechanical stress, parasitism, and the demands of embryonic gas exchange [20,126,127]. As the chorion constitutes the outermost eggshell layer and the primary interface between the embryo and the external environment, its components are likely exposed to stronger ecological pressures than proteins of inner layers such as the vitelline membrane. Consistent with this hypothesis, comparative analyses in *Drosophila* have demonstrated that chorion proteins evolve more rapidly than vitelline membrane proteins [20], while studies in *B. mori* have revealed accelerated evolution of middle chorion genes [93]. Evolutionary diversification is also reflected in the organization of chorion gene repertoires across insect lineages. For example, *B. mori* possesses large multigene chorion clusters [93], *Drosophila* species employ locus-specific chorion gene amplification to achieve high transcriptional output [79], and mosquitoes have evolved lineage-specific eggshell proteins associated with specialized chorion functions [128,129]. Functional specialization is further manifested in the structural properties of chorion proteins. Glycine- and proline-rich proteins can contribute to matrix assembly and flexibility, similar to what happens with mammalian proteins such as elastin [130], whereas tyrosine-rich proteins provide substrates for oxidative crosslinking reactions that promote chorion hardening and stabilization [35,131,132]. Likewise, vitelline membrane proteins establish the initial scaffold upon which subsequent eggshell layers are deposited, while enzymes such as peroxidases and laccases mediate the maturation and stabilization of the chorion matrix through post-translational crosslinking processes [129,133,134]. Collectively, these observations indicate that ecological adaptation has shaped both the evolutionary trajectories of chorion gene families and the structural characteristics of their encoded proteins, ultimately contributing to the remarkable diversity of eggshell architectures observed across insects.

## 4. Chorion Composition

The insect chorion is a complex extracellular structure whose composition varies considerably among species, reflecting differences in reproductive strategies and environmental adaptations. Proteomic studies have revealed that the chorion is predominantly composed of structural proteins, although the identity, abundance, and diversity of these proteins can differ markedly across taxa [135,136]. Following their secretion, these proteins often undergo extensive post-translational modifications, including crosslinking processes, frequently associated with peroxidase-mediated reactions and tanning, that contribute to mechanical stabilization and protection against desiccation [35].

**Figure 4:**
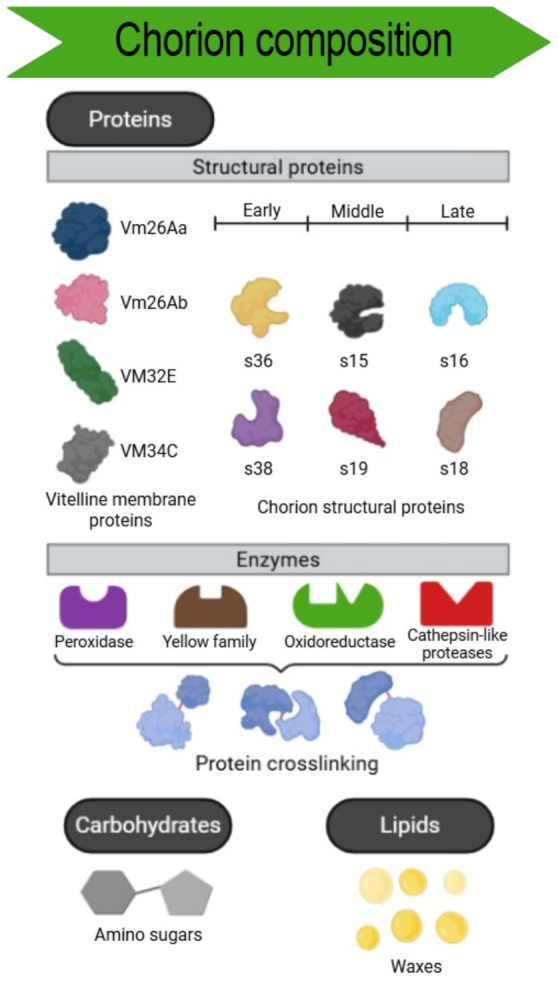
Biochemical composition of *D. melanogaster* chorion and its contribution to eggshell maturation. Schematic overview of the principal molecular components that constitute the insect eggshell. The chorion is predominantly composed of structural proteins, including vitelline membrane proteins and chorion structural proteins. Following secretion, these proteins undergo extensive post-translational modifications, particularly oxidative crosslinking reactions mediated by enzymes such as peroxidases, oxidoreductases, members of the yellow protein family, and proteases, resulting in the stabilization and maturation of the eggshell matrix. In addition to proteins, the chorion may contain carbohydrates, including amino sugar-containing compounds that contribute to matrix organization and mechanical properties. Lipid components, particularly waxes, are incorporated into inner eggshell layers and form hydrophobic barriers that reduce water loss and enhance resistance to environmental stress. Although the relative abundance and diversity of these components vary considerably among insect taxa, their combined interactions determine the mechanical integrity, permeability, and protective functions of the mature chorion.

In addition to its proteinaceous matrix, the chorion may also incorporate other biomolecules such as carbohydrates, which can contribute to structural organization and rigidity, although their presence and functional significance remain variable among insect groups. Plus, lipid components, including hydrocarbons and waxes, have been identified in the inner layers of the chorion in several species, where they form hydrophobic barriers that enhance resistance to water loss and environmental stress (Figure 4) [35,135,136].

The extent, origin, and functional integration of these different components are not uniformly understood across insects. Therefore, this section will synthesize current knowledge on the protein, carbohydrate, and lipid composition of the chorion, with emphasis on the species in which these components have been characterized.

### 4.1. Chorion Protein Composition

The protein composition of the insect chorion constitutes the fundamental structural basis of the eggshell and has been investigated in some species. In general, the chorion is predominantly composed of a diverse array of structural proteins secreted by follicle cells during choriogenesis, which assemble into a highly organized extracellular matrix. Proteomic and molecular analyses have revealed that the diversity and relative abundance of these proteins can vary significantly among insect taxa, contributing to species-specific differences in eggshell architecture, permeability, and mechanical properties [136,137,138]. Proteomic and molecular studies in different insect species have provided detailed insights into the composition and organization of chorion proteins. In the model organisms *D. melanogaster* and *B. mori*, the chorion proteome has been characterized, revealing large multigene families encoding structurally related proteins that are expressed in a tightly regulated temporal sequence during choriogenesis, as mentioned earlier [137,139,140,141]. In contrast, studies in other dipteran species, including mosquitoes such as *A. aegypti* [128,129] and *Anopheles gambiae* [127], have highlighted both conserved features and species-specific adaptations in chorion composition, particularly in relation to environmental challenges such as desiccation resistance.

In *D. melanogaster*, the chorion constitutes one of the best-characterized extracellular protein matrices in insects. These proteins are classically categorized into early, middle, and late chorion proteins, such as s36, s38, s15, s19, s16, and s18, which are sequentially expressed during late oogenesis under a tightly regulated temporal program [81,139]. In addition to these major components, a number of minor chorion-associated proteins have also been identified, including putative structural and regulatory proteins, as well as enzymes (yellow family, peroxidase, oxidoreductase, cathepsin-like proteases) likely involved in matrix crosslinking [139]. Many chorion proteins are relatively small and enriched in proline, alanine, and glycine residues, and frequently contain repetitive sequence motifs that facilitate intermolecular interactions and higher-order assembly [137]. Following their secretion by follicle cells, these proteins undergo extensive post-translational modifications, including oxidative crosslinking reactions, which contribute to the formation of a mechanically robust and functionally impermeable eggshell structure [81,137,138,139].

The functional characterization of chorion proteins in *B. mori* remains comparatively limited. In this species, as well as in other Lepidoptera, the chorion is composed of a highly complex and structurally organized set of proteins encoded within a single, continuous genomic locus [93]. Chorion proteins in *B. mori* are characterized by a modular organization comprising a highly conserved central domain, which is homologous within and between gene families of the same evolutionary branch, flanked by more variable amino- and carboxy-terminal regions (“arms”). These terminal domains are enriched in tandemly repetitive peptide motifs, in contrast to the central domain where such repeats are not evident, and are thought to contribute to intermolecular interactions, ordered assembly, and crosslinking during chorion [91,142]. These proteins are generally small and exhibit low solubility, a property likely associated with their high content of glycine and other hydrophobic amino acids, including alanine and leucine [140]. Proteomic analyses have shown that not all predicted chorion proteins are detectable at the protein level, suggesting that they may be present at very low levels, or that they may be derived from pseudogenes containing multiple stop codons that do not yield detectable peptides or even difficulty in extraction [93]. Notably, such analyses have also provided evidence for spatially localized expression of specific chorion components, as certain proteins were detected exclusively in the micropyle region. This observation, derived from comparative proteomic analyses of dissected eggshell regions with and without the micropyle, indicates a degree of regional specialization in chorion composition that likely reflects functional differentiation within the eggshell [93].

In mosquitoes such as *A. aegypti*, the chorion proteome reflects specific adaptations that enhance egg survival under fluctuating environmental conditions, particularly resistance to desiccation [143]. Proteomic analyses have identified a complex repertoire comprising more than 200 proteins [129]. In addition to structural components, the eggshell contains a diverse array of enzymes, including proteases, chitinases, odorant-binding proteins, peroxidases, transglutaminases, prophenoloxidases, phenoloxidases, dopachrome-converting enzymes (including members of the yellow protein family), and laccase-like multicopper oxidases [128,129]. Furthermore, a subset of chorion proteins is enriched in cysteine residues and hydrophobic regions rich in proline, alanine, valine or leucine, which likely contribute to protein stability and intermolecular crosslinking [128,131]. This molecular profile indicates a coordinated involvement of biochemical pathways associated with crosslinking, melanization, and sclerotization, ultimately promoting increased chorion rigidity, reduced permeability, and enhanced protection against environmental stressors [128,129,131].

In contrast to *A. aegypti*, *An. gambiae* produces eggs with a more permeable chorion and reduced resistance to desiccation, with embryonic development typically occurring in humid or aquatic environments. This contrast highlights that even closely related taxa can exhibit substantial differences in eggshell properties, reflecting distinct reproductive strategies and ecological adaptations. Proteomic analyses in *An. gambiae* have identified a set of candidate eggshell-associated proteins, including both vitelline membrane and chorion structural components. In addition to these structural proteins, several enzymes have been detected, such as phenoloxidases, laccases, dopachrome-converting enzymes, prophenoloxidases, and peroxidases, suggesting the involvement of conserved biochemical pathways related to chorion maturation and stabilization. Despite these advances, the molecular composition of the *Anopheles* chorion remains comparatively less well characterized, and current evidence suggests a lower degree of complexity relative to that described in other insect models [127].

The hemipteran *Diaphorina citri*, known as the Asian citrus psyllid, is a sap-sucking and main vector for *Candidatus* Liberibacter asiaticus, the causal agent of citrus huanglongbing (HLB). In this species, the chorion proteome has been characterized through mass spectrometry-based approaches, revealing a moderately complex protein composition compared to other insect models. Proteomic analyses identified a total of 51 chorion-associated proteins, which were functionally classified into multiple categories, including enzymes, binding proteins, structural components, homeostasis-related proteins (predominantly vitellogenins), proteins associated with gene expression, immune-related proteins, other proteins and a substantial proportion of uncharacterized proteins. Notably, enzymes represent the most abundant group, accounting for approximately 25% of the identified proteins, and include hydrolases, peptidases, and glycoside hydrolases such as chitinases and glucosidases, suggesting roles in structural remodeling and chitin metabolism. The presence of binding proteins, including chitin-binding and odorant-binding proteins, was also shown [144].

Comparative analyses across *D. melanogaster*, *B. mori*, mosquitoes, and hemipterans such as *D. citri* reveal that differences in protein repertoire, abundance, structural organization, and associated enzymatic components give rise to distinct physicochemical properties of the eggshell. These variations are evident not only between distantly related insect orders but also among closely related species, such as *A. aegypti* and *An. gambiae*, where contrasting chorion compositions are associated with divergent ecological requirements and reproductive strategies. Thus, while a conserved functional framework underlies chorion formation, the specific molecular composition of the eggshell reflects lineage-specific adaptations, indicating that the chorion should be regarded as a highly specialized and evolutionarily flexible structure whose properties are fine-tuned to the biological and environmental context of each species [127,128].

#### Biochemical Mechanisms of Chorion Stabilization and Maturation: Crosslinking and Tanning

Post-translational modifications play a central role in the functional maturation of the insect chorion, particularly through biochemical processes such as protein crosslinking, tanning, and sclerotization [35,145]. Following secretion by follicle cells, chorion proteins undergo biochemical maturation processes enzymatically mediated that promote the formation of covalent bonds, leading to increased structural rigidity, reduced solubility, and enhanced resistance to environmental stressors [145]. These processes are typically driven by oxidative mechanisms involving enzymes such as peroxidases, laccases, and phenoloxidases, which catalyze the formation of dityrosine and other crosslinked protein networks [145,146]. In addition, tanning reactions, often associated with catecholamine metabolism, contribute to pigmentation, hardening, and waterproofing of the eggshell. Together, these mechanisms are essential for establishing the mechanical integrity and protective properties of the chorion, ensuring embryo survival under diverse environmental conditions [145,146].

Comparative studies across insect taxa indicate that, although the fundamental biochemical principles underlying chorion hardening are broadly conserved, the specific mechanisms and enzymatic pathways involved vary among species. In mosquitoes such as *A. aegypti*, chorion hardening is strongly associated with peroxidase-mediated crosslinking of structural proteins, resulting in the formation of disulfide and dityrosine bonds that render the eggshell insoluble and resistant to desiccation [131,147]. This process is further complemented by phenoloxidase and dopa decarboxylase-driven melanization, which enhances chorion impermeability and environmental resistance [148,149]. It is important to emphasize, as previously discussed, that there is substantial variation in how eggs from different mosquito species respond to distinct environmental conditions. In this context, the degree of chorion melanization emerges as a key determinant of desiccation resistance. Eggs of *Anopheles aquasalis* and *Culex quinquefasciatus*, which exhibit lower levels of melanization, show reduced tolerance to dry conditions when compared to those of *A. aegypti*. Consequently, embryonic viability tends to be significantly higher in species with darker eggshells than in those with lighter-colored chorion [150]. Besides, this mechanism also appears to be important in *A. gambiae*, as the inhibition of DOPA decarboxylase reduced melanization and the hatching rate of the eggs [151].

Across different dipteran species, both similarities and distinctions can be observed when compared to mosquitoes with respect to chorion maturation processes. In *D. melanogaster*, tanning mechanisms appear to be less pronounced, as the eggshell remains largely unpigmented and does not undergo significant melanization [145]. Nevertheless, chorion stabilization is primarily achieved through peroxidase-dependent crosslinking reactions involving hydrogen peroxide, leading to the formation of di- and tri-tyrosine bonds [134,152,153]. This oxidative crosslinking mechanism is consistent with that described in other dipterans, such as the olive fly *Bactrocera oleae* [154,155], indicating a conserved biochemical strategy for eggshell stabilization despite differences in pigmentation and tanning intensity.

In the eggshell of the almond seed wasp *Eurytoma amygdali*, histochemical assays have demonstrated the presence of peroxidase activity within the chorion, from which a potential role in modulating chorion elasticity has been inferred, possibly through the formation of resilin-type crosslinks [156]. In the hemipteran *R. prolixus*, chorion hardening involves a tightly regulated oxidative system in which hydrogen peroxide production, mediated by ovarian dual oxidase (Duox), a member of the NADPH oxidase family, drives dityrosine-based protein crosslinking. This process is essential for the mechanical stabilization and proper hardening of the eggshell [132]. In moths of the genus *Hyalophora*, the chorion is hardened by disulfide crosslinks between cysteine-rich proteins, rather than by polyphenol oxidase tanning [29].

### 4.2. Chorion Carbohydrate Composition

Carbohydrates in the insect chorion are generally present as minor constituents relative to proteins, and quantitative data on total carbohydrate content in the chorion remain extremely scarce and highly variable among taxa; nevertheless, they are likely to play important structural and functional roles. In *Aedes* mosquitoes, the presence of carbohydrates has been detected in solubilized chorion preparations used for protein analysis [157]. Furthermore, a chitin-like carbohydrate has been identified in the eggshell of *A. aegypti* [158]. This polysaccharide, a polymer of N-acetylglucosamine, is a prominent component of insect extracellular matrices, particularly the cuticle (exoskeleton) [159] and serosal cuticle [160]. In species such as *A. aegypti*, *Anopheles* spp., and *Culex* spp., chitin deposition in the extraembryonic serosal cuticle has been directly associated with increased desiccation resistance, suggesting that even relatively small amounts of carbohydrate polymers can have profound ecological and physiological implications [160,161].

In addition to chitin, carbohydrates are also present in the form of glycoproteins integrated into the chorion matrix. These molecules may contribute to chorion assembly, cross-linking, and functional specialization, such as chorion peroxidase, a known glycoprotein that catalyzes protein cross-linking during chorion hardening [162]. In *D. melanogaster*, chorion and vitelline membrane contain small amounts of carbohydrates, mainly amino sugars, which account for approximately 6% of the chorion [137]. In members of the order Orthoptera, such as the grasshopper *Oxya hyla hyla*, histochemical assays revealed a positive PAS reaction in the chorion layer, suggesting the presence of a carbohydrate fraction, possibly amino sugars [163]. In another grasshopper, *Gesonula punctifrons*, the chorion is formed by protein fibers intercalated with polysaccharides, indicating a protein–polysaccharide matrix [164].

### 4.3. Chorion Lipid Composition

Lipids represent an additional and functionally critical class of molecules contributing to eggshell structure and physiology. Although generally present in lower abundance compared to structural proteins, lipids, particularly hydrocarbons and waxes, play essential roles in modulating eggshell permeability and environmental resistance [165,166]. These lipids are typically associated with specialized interfaces such as the vitelline envelope, where they contribute to the formation of hydrophobic barriers that limit water loss and protect the developing embryo against desiccation [35].

A well-documented manifestation of lipid incorporation into the insect eggshell is the formation of a wax layer, which is sometimes, but not universally, present on the external surface of the vitelline envelope. This structure has been described in multiple insect groups, including species of *Drosophila* and other dipterans, *R. prolixus*, grasshoppers, and some Lepidoptera, suggesting that it may be a widespread feature, particularly among species whose eggs are exposed to desiccating conditions [138]. Ultrastructural analyses in *D. melanogaster* have demonstrated that the wax layer forms a hydrophobic interface located between the vitelline membrane and the crystalline intermediate chorionic layer. This structure originates from the accumulation and subsequent fusion of lipid-filled vesicles secreted by follicle cells, ultimately giving rise to a water-impermeable barrier [36,165,167,168]. A comparable waxy layer has also been described in *Drosophila grimshawi*, where it is presumed to perform a similar function in regulating eggshell permeability [169].

Beyond the genus *Drosophila*, lipid-associated barriers have been identified in a wide range of insect taxa. In dipterans such as *Lucilia cuprina* [170], hemipterans including *R. prolixus* [166,171], lepidopterans such as *Diatraea saccharalis* [172] and *Manduca sexta* [16], and orthopterans like *Melanoplus differentialis* [173] and *Eyprepocnemis plorans* [174], the presence of a wax layer or wax-like material has been implicated in the control of eggshell permeability, supporting a broadly conserved role for lipids in eggshell physiology. Additionally, hydrocarbon compounds have been detected in the vitelline membrane of several dipteran species, including *Musca domestica*, *Cochliomyia hominivorax*, *Cochliomyia macellaria*, *Phaenicia sericata*, *L. cuprina*, and *Anastrepha ludens* [175], further highlighting the diversity of lipid constituents associated with the insect eggshell.

## 5. Chorion Structure

The chorion matrix is not a homogeneous structure but rather a highly organized extracellular matrix exhibiting complex three-dimensional morphology at both the surface and internal levels. At the macroscopic and ultrastructural levels, the chorions from different species display distinct external surface patterns and a multilayered internal architecture, which together determine properties such as permeability, mechanical resistance, and environmental interaction. Therefore, understanding chorion structure requires examining both surface morphology and cross-sectional organization, as well as the specialized regions that confer functional versatility to the eggshell [136].

### 5.1. General Structural Organization and Specialized Features of the Chorion

**Figure 5:**
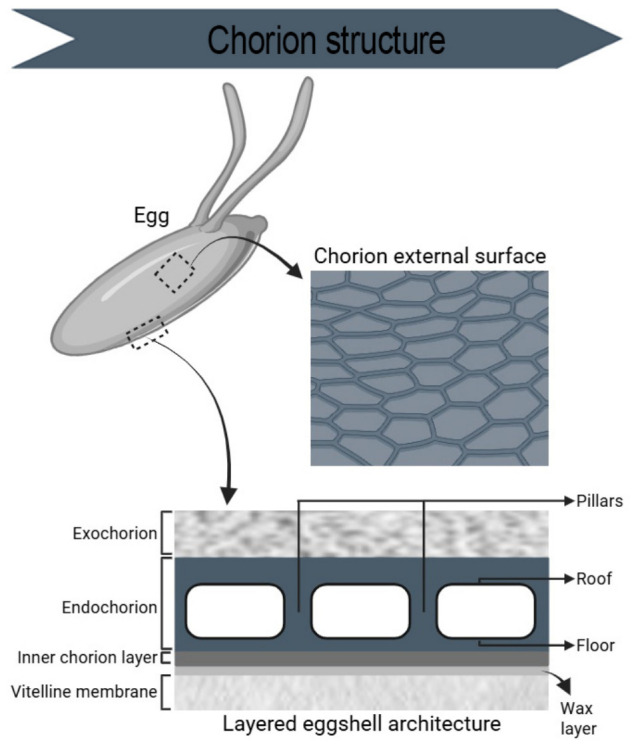
General organization of the insect eggshell and its multilayered chorion architecture. Schematic representation of the hierarchical organization of the *D. melanogaster* eggshell, illustrating both its external surface patterning and cross-sectional architecture. The eggshell is composed of two principal components: an inner vitelline membrane, which constitutes the first layer deposited around the oocyte, and an outer chorion. The chorion is typically organized into multiple layers, including an inner chorion layer, the endochorion, and the exochorion. The endochorion commonly exhibits a complex internal architecture composed of specialized sublayers such as the floor, pillar, and roof regions, which contribute to the mechanical support and spatial organization of the eggshell. The exochorion forms the outermost chorion layer and gives rise to the surface ornamentation visible on the egg exterior. A wax-rich layer is associated with the inner chorion region and contributes to the formation of a hydrophobic barrier that limits water loss. It is worth noting that the number, thickness, and ultrastructural organization of the layers can vary considerably among insect taxa, but in general, the basic hierarchical structure of multiple layers builds the remarkable diversity of insect eggshell architectures.

**Figure 6:**
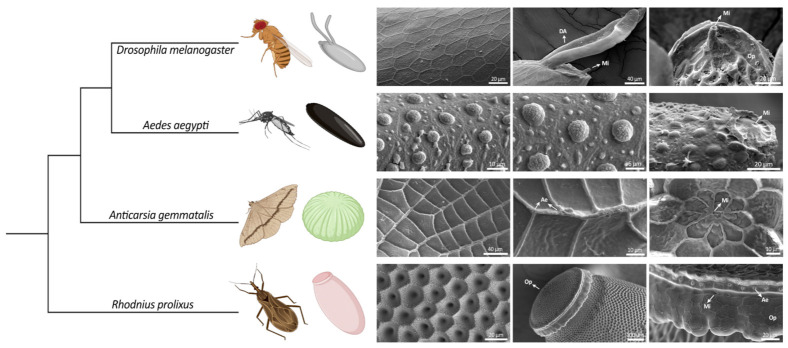
The external surface of the insect chorion displays remarkable morphological diversity across taxa, reflecting extensive evolutionary divergence in eggshell architecture and specialization. Representative scanning electron micrographs illustrate distinct chorionic ornamentation patterns, aeropylar structures, micropyles, and overall eggshell organization in four insect species belonging to different orders. Notably, substantial differences are observed even between closely related taxa, such as the dipterans *D. melanogaster* and *A. aegypti*. While *D. melanogaster* presents a polygonal chorionic pattern associated with prominent dorsal appendages, *A. aegypti* exhibits a highly ornamented exochorion composed of tuberculate structures distributed throughout the eggshell surface. Likewise, Lepidoptera and Hemiptera display species-specific chorionic architectures characterized by distinct geometric patterns, aeropyles, and opercular specializations. These variations highlight the extraordinary structural plasticity of the insect chorion and reinforce the idea that eggshell morphology is shaped by phylogenetic history, developmental processes, reproductive strategies, and ecological adaptations. Abbreviations: Ae, aeropyle; DA, dorsal appendages; Mi, micropyle; Op, operculum.

In general, the insect eggshell consists of two principal layers: an inner vitelline membrane, which lies in direct contact with the oocyte and forms a thin and relatively uniform layer, as mentioned earlier, providing an initial protective interface and contributing to selective permeability, and an outer chorion [35,136,176]. The chorion itself exhibits a complex multilayered organization, typically comprising successive chorionic layers that are broadly subdivided into an inner endochorion and an outer exochorion, each of which may be further differentiated into structurally and functionally distinct sublayers (Figure 5) [35]. Ultrastructural analyses across diverse insect taxa have shown that these layers are arranged into highly elaborate architectures, often involving lamellar arrays, pillar-like elements, and aeropylar channels that collectively contribute to mechanical stability and gas exchange. The external surface of the chorion displays remarkable morphological diversity (Figure 6), ranging from relatively smooth to highly ornamented textures. These surface patterns frequently include species-specific features such as reticulations, ridges, and polygonal imprints, which are thought to reflect the spatial organization of follicular epithelial cell interfaces during chorion deposition. Such morphological specializations may be associated with underlying cellular templates and are likely to play roles in adhesion, camouflage, and interactions with the surrounding environment [17,35,136,176,177].

In addition, the chorion comprises a range of specialized structures that are essential for egg function. Among these, the micropyle represents a conserved feature that forms a channel for sperm entry during fertilization, as mentioned earlier, typically located at the anterior pole and exhibiting considerable structural diversity across insect taxa [178]. Aeropyles, in turn, are pore-like structures connected to the egg interior through chorion-traversing canals. Their number, distribution, and structural complexity vary among species and are often associated with ecological conditions. These structures may be distributed across the entire chorion surface or restricted to specific regions, where they facilitate gas exchange between the developing embryo and the external environment, ensuring adequate oxygen supply while maintaining protective functions [179,180,181]. Another key feature is the operculum, a specialized cap-like structure typically located at the anterior pole and delineated by a hatching line, which enables the controlled emergence of the larva or nymph. The mechanical properties of the operculum are developmentally regulated, such that the force required for its opening generally decreases as embryogenesis progresses. Notably, micropyles and aeropyles are frequently associated with this anterior region. In certain species, additional specialized structures, such as dorsal appendages, plastrons (specialized water-repellent surface structures, often meshwork or hairs that act as physical gills, allowing eggs to breathe while submerged), or other respiratory adaptations, further enhance gas exchange, as previously mentioned, particularly in aquatic or humid environments [35].

### 5.2. Diversity of Chorion External Surface Morphology Among Insect Taxa

Among dipterans, the chorion surface exhibits some of the most extensively characterized patterns of morphological diversification. The family Drosophilidae provides one of the most informative models, revealing extensive ultrastructural variation. In *D. melanogaster*, the eggshell surface is characterized by a highly ordered exochorion displaying a regular polygonal (often hexagonal) pattern that reflects the spatial arrangement of follicular epithelial cells during secretion. The micropyle forms an open-ended, hollow conical structure, surrounded by a specialized region known as the collar, which ensures efficient sperm entry [167]. Comparative studies across drosophilid species, including Hawaiian lineages and *Scaptomyza* taxa, reveal substantial diversity in chorion morphology. While the polygonal surface pattern is generally conserved, its regularity and sculpturing vary widely. Dorsal appendages represent a major axis of variation, differing in number, size, shape, and complexity, from the typical pair in *D. melanogaster* to multiple, elongated, or even absent structures in other species, often reflecting adaptation to specific oviposition environments. These appendages function in respiration, and their diversification is closely tied to ecological conditions. Additional variation occurs in the micropyle and aeropylar systems, which differ in structure, density, and distribution, influencing chorion permeability and gas exchange efficiency [20,182,183].

In mosquitoes, chorion morphology reflects ecological adaptation, particularly to oviposition sites and desiccation resistance. Eggs of *A. aegypti* display a highly ornamented exochorion with well-defined polygonal networks and complex tubercle patterns, contributing to surface heterogeneity and likely affecting permeability and mechanical properties. The micropylar apparatus is also distinctive, with a prominent collar and radially arranged tubercles. Comparative studies across *Aedes* species reveal substantial variation in exochorionic ornamentation and micropylar structure, ranging from highly organized, elevated configurations to more irregular and diffuse forms [184,185,186,187,188]. In contrast, eggs of *Anopheles* show distinct morphology adapted to aquatic oviposition. Instead of extensive polygonal ornamentation, the chorion features lateral float structures that provide buoyancy. The exochorion is asymmetric, with more prominent, star-shaped dorsal tubercles and less pronounced, interconnected ventral protuberances. The micropyle is positioned anteriorly on the ventral side but is less elevated and more integrated into the chorion, lacking the prominent collar seen in culicine mosquitoes like *A. aegypti* [189].

Phlebotomine sandflies (Diptera: Psychodidae) display chorion morphologies that are highly species-specific and taxonomically informative. Unlike the regular polygonal patterns of drosophilids or the pronounced ornamentation of mosquitoes, their eggs exhibit diverse sculpturing, including polygonal networks, parallel ridges, and more complex forms such as volcano-like or placoid structures, reflecting differences in chorion deposition. Variation also occurs in the micropyle and aeropylar systems, though these are generally less prominent. The micropyle is typically anterior and subtly integrated into the chorion, while aeropyles are often embedded within the surface architecture, linking chorion structure to respiratory function [190,191].

In Lepidoptera, chorion morphology is typically highly ordered and symmetrical, featuring radial ridges, polygonal follicle cell imprints, reticulated networks, aeropyles, and a rosette-shaped micropylar apparatus, though interspecific variation is common. In *B. mori*, the chorion is regularly organized, with radially extending aeropyles and a prominent anterior micropyle rosette, usually with a single opening. Comparisons with *Bombyx mandarina* reveal differences in micropyle arrangement and the presence of larger, irregular knob-like structures within the polygonal pattern [192,193]. Across Lepidoptera more broadly, variation in micropyle number and organization represents a major axis of structural diversification. For example, in species such as *M. sexta* and *Sesamia nonagrioides*, the micropyle region also consists of a rosette containing a single micropylar opening [194], similar to that observed in *Ephestia kühniella* [46]. In contrast, studies on other pest species such as *Cydia pomonella*, *Heliothis virescens*, *Spodoptera littoralis*, *Anticarsia gemmatalis*, *Chrysodeixis includens*, *Rachiplusia nu*, *Spodoptera albula*, *Spodoptera cosmioides*, *Spodoptera eridania*, *Spodoptera frugiperda*, *Helicoverpa armigera*, *Helicoverpa gelotopoeon*, and *Chloridea virescens* have revealed variation in both micropyle number, typically ranging from two to six, and in the degree of rosette prominence. In these species, the micropylar rosette may be positioned on a raised micropylar cone, from which canals open in the micropylar plate, or it may appear more diffuse, forming less elevated micropylar regions that are continuous with the general contour of the egg surface [195,196]. Beyond the micropyle, chorion surface ornamentation also shows marked diversity. In *Euproctis chrysorrhoea*, most of the egg surface, except for the micropylar region, appears smooth and lacks prominent features other than aeropyles [197]. In contrast, in the castor butterfly *Ariadne merione*, the surface bears longitudinal ridges, grooves, protuberances, and several specialized respiratory filaments with terminal aeropyles, suggesting a close relationship between structural patterning and respiratory function [198]. In *Nymphula nymphaeata*, a species adapted to aquatic or semi-aquatic environments, the chorion lacks aeropyles entirely [199].

Hemipteran eggs display remarkable diversity in chorion surface morphology, closely associated with their varied reproductive strategies and ecological niches. In Triatominae, particularly in species of the genus *Rhodnius* (e.g., *R. prolixus*, *Rhodnius neglectus*, *Rhodnius domesticus*, *Rhodnius colombiensis*, *Rhodnius milesi*, and *Rhodnius stali*), the chorion typically exhibits a pattern of depressions with irregular pentagonal or hexagonal shapes, resulting from follicular cell imprints. A conspicuous operculum is present at the anterior pole, which in some species forms a collar-like structure along its border. This opercular region is functionally complex, housing both micropyles and aeropyles, which generally occur as simple pore-like openings [19,179,200,201]. In other reduviids, such as species of *Belminus*, a well-defined polygonal pattern is restricted primarily to the opercular region, which also bears globular projections, whereas the remaining chorion surface appears comparatively smoother. As in *Rhodnius*, both aeropyles and micropyles are concentrated in the opercular area [202]. Among Cimicidae, *Cimex lectularius* exhibits a chorion covered by polygonal projections across the entire egg surface. The opercular region is differentiated into three distinct surface types: (i) larger, distinctly striated and rough polygons; (ii) polygons bearing spherical structures; and (iii) smooth polygons [203]. In contrast, in another cimicid called *Ornithocoris pallidus*, the exochorion presents prominent spherical or polygonal relief structures over the entire egg surface, and pseudomicropyles are found along the opercular margin [204]. In phytophagous hemipterans such as *Nezara viridula* (Pentatomidae), the eggs are typically barrel-shaped and coated with a specialized adhesive secretion produced by follicular cells, forming a cement-like layer that modifies the external surface and facilitates attachment to plant substrates, while potentially influencing chorion permeability. The opercular region displays extensive ornamentation, including conical projections and mushroom-shaped structures, the latter likely contributing to water repellency, which are interconnected by ridges and exhibit a granular apical surface. From this region also arise clavate aero-micropylar processes [205]. More extreme structural specializations are observed in aquatic or semi-aquatic taxa. In the giant water bug *Abedus herberti* (Belostomatidae), the chorion possesses highly specialized respiratory adaptations, including plastron surfaces and elaborate aeropylar systems that maintain a thin air layer, enabling efficient gas exchange under submerged or high-humidity conditions [206]. Similarly, in the invasive planthopper *Lycorma delicatula* (Fulgoridae), the egg surface is composed predominantly of interconnected hexagonal plates forming a plastron-like network with regionally complex sculpturing. The operculum is spheroidal and surrounded by a porous aeropylar network, and a putative micropylar process forms a stem-like nodule with a concave, flower-like base [207].

In Orthoptera, chorion surface morphology is generally associated with oviposition in soil or protected terrestrial substrates, resulting in relatively robust eggshells with adaptations that emphasize mechanical protection and controlled gas exchange. In acridid grasshoppers such as *Locusta migratoria*, the chorion exhibits comparatively low external ornamentation. Rather than presenting highly elaborate surface projections, the eggshell typically displays subtle sculpturing, including dotted patterns or shallow depressions, with a predominance of minute protuberances and no clearly defined polygonal pattern derived from follicular cell imprints. In the micropylar region, unusually located near the posterior pole, this sculpturing is absent [208]. In contrast, in species such as *G. punctifrons*, the eggshell exhibits a more distinctly sculptured surface, with the entire chorion covered by a pentagonal and hexagonal architecture. At the posterior pole, a cap-like structure is present, likely associated with fertilization at this site [164]. Comparative analyses across multiple grasshopper species further demonstrate that chorion surface sculpturing can vary considerably, ranging from regular to irregular polygonal networks, reticulated designs with feather-like projections, or even more rounded surface patterns [209]. In Tettigoniidae, such as *Isophya nervosa*, the micropylar region is located at the anterior pole and comprises approximately 8–12 micropyles, which are slightly elevated relative to the egg surface and arranged in a circular pattern. The chorion surface exhibits a polygonal, often pentagonal pattern, with unequal side lengths, and contains tubular air channels interpreted as aeropyles, typically appearing as rounded to oval openings within hexagonal units [210]. Scanning electron microscopy studies of Rhaphidophoridae (cave Orthoptera) eggs have revealed that the outer chorion surface exhibits a highly organized architecture composed of polygonal fields delimited by raised ridges, which vary in size and prominence among species. Aeropyles are widely distributed across the egg surface, opening externally as small pores. In these taxa, micropyles are not located at the anterior pole but instead occur in the equatorial region, where they are organized into a characteristic rosette-like structure [211].

A compilation of representative studies addressing the external morphology of the chorion in different insect species is provided in Table S2.

### 5.3. Cross-Sectional Architecture and Layered Organization of the Chorion Among the Insect Taxa

Across insect taxa, differences in cross-sectional architecture extend beyond general layer composition to encompass the fine-scale organization and interaction of internal structural elements. Comparative ultrastructural studies indicate that the relative prominence and internal configuration of chorionic substructures can vary considerably among groups. In many species, the endochorion exhibits a more complex internal arrangement, often incorporating trabecular networks, columnar elements, or heterogeneous matrices that enhance mechanical resilience and structural integration. In contrast, the exochorion shows pronounced variability in its degree of compaction and continuity, ranging from tightly consolidated outer layers to more porous or regionally differentiated structures linked to external specializations. Variability is also evident in the architecture of pore systems, whose density, orientation, and connectivity across layers differ substantially among taxa, with direct implications for gas exchange efficiency [35].

In Diptera, the cross-sectional architecture of the chorion has been most comprehensively characterized in *D. melanogaster*, where the eggshell exhibits a highly stratified and regionally specialized organization. The chorion is composed of multiple distinct layers arranged sequentially from the oocyte outward: (i) the vitelline membrane, consisting of irregularly organized particles; (ii) a wax layer composed of hydrophobic plates that split tangentially; (iii) an innermost chorionic layer characterized by a crystalline structure; (iv) the endochorion consisting of a fenestrated basal layer, a network of vertically oriented pillar-like elements, and a more compact outer roof layer; and (v) the exochorion, the outermost and relatively thinnest layer, that consists of loose fibers that tend to be oriented parallel to the oocyte surface and which gives rise to the surface specializations of the eggshell. Each of these layers exhibits distinct ultrastructural properties. The endochorion represents the principal structural component and is organized into a characteristic tripartite floor–pillar–roof arrangement. This configuration provides mechanical stability while maintaining low density. In contrast, the exochorion primarily contributes to the formation of external surface features, whereas the underlying wax layer plays a key role in conferring impermeability. Notably, this layered architecture is not uniform across the eggshell; regional variations occur in areas such as the micropylar region and dorsal appendages, reflecting localized differences in follicular cell activity during chorion deposition [79,167,212,213]. In other fly species, including *Ceratitis capitata*, *Dacus oleae*, *Megaselia scalaris*, *Megaselia spiracularis*, as well as the closely related *D. grimshawi*, the overall cross-sectional architecture of the chorion remains broadly comparable to that of *D. melanogaster*, with conservation of the canonical five-layered organization, including the characteristic tripartite arrangement of the endochorion. However, fine-scale ultrastructural differences are consistently observed, primarily involving variations in layer thickness and the presence or absence of specific features, such as aeropylar canals traversing the outer endochorion, which are not present in *D. melanogaster* [169,214,215,216].

In mosquitoes, such as species of *Aedes*, *Anopheles*, *Culex*, and *Toxorhynchites*, the tripartite pillar-based organization typical of other model dipterans is not observed, and the chorion displays a comparatively simpler internal architecture. Culicidae exhibit a relatively conserved chorion organization, generally consisting of three main layers: (i) the vitelline envelope, (ii) the endochorion, and (iii) the exochorion. Notably, unlike in *Drosophila* and other flies, a distinct and well-defined wax layer is not consistently recognized as a separate structural component of the chorion. The vitelline envelope is typically homogeneous and lacks evident substructural complexity, whereas the endochorion represents the primary structural component, always exhibiting a lamellar organization and the presence of tubercles of varying size and shape (except in *Toxorhynchites*), that contribute to mechanical support. In contrast, the exochorion is thinner and more variable, forming a continuous or loosely organized outer layer that covers the underlying endochorionic tubercles. Despite this overall structural conservation, important intergeneric variations are evident. For instance, in *Anopheles*, the exochorion may be absent in specific regions, such as the ventral surface, whereas in *Toxorhynchites*, the distinction between endochorion and exochorion becomes less defined, with partial fusion of layers and the presence of internal voids. In *Culex pipiens*, the exochorion is further specialized, mediating adhesion between adjacent eggs in raft formations, while the endochorion consists of numerous tubercles of varying size. Compared to *D. melanogaster*, which exhibits a highly differentiated, pillar-supported endochorion (floor–pillar–roof system), the chorion of mosquitoes is thus characterized by reduced internal compartmentalization and a greater emphasis on functional specializations related to environmental interaction, such as flotation, adhesion, and resistance to desiccation [217,218,219,220].

In Lepidoptera, the cross-sectional architecture of the chorion generally conforms to a conserved structural framework characterized by a multilayered organization comprising a compact, lamellar exochorion and an underlying, more loosely organized trabecular endochorion, both of which are supported internally by the vitelline membrane. This innermost layer lies in direct contact with the oocyte and forms a thin, relatively homogeneous interface that contributes to selective permeability and structural continuity. Across diverse species, including *B. mori*, *Utetheisa ornatrix*, *C. pomonella*, *H. virescens*, *S. littoralis*, *Plodia interpunctella*, *Amyelois transitella*, *E. kuehniella*, *Corcyra cephalonica*, and *A. gemmatalis*, the chorion is frequently described using a hierarchical subdivision into C1, C2, and C3 layers, reflecting differences in ultrastructural organization and electron density. Within this framework, the C1 layer corresponds to the innermost chorionic region; the C2 layer represents an intermediate, trabecular zone supported by trabeculae and connected to the exterior via aeropylar canals; and the C3 layer corresponds to the outermost, highly compact and often lamellar exochorion. The exochorion (C3) consistently represents the most electron-dense and mechanically robust component, often exhibiting a lamellar or helicoidal arrangement of proteinaceous fibers that confer resistance to environmental stress. Beneath this, the endochorion (C1–C2) is generally composed of a trabecular or fibrillar matrix, forming a three-dimensional porous network that provides structural support while facilitating internal connectivity and gas diffusion. In many cases, aeropylar canals traverse these layers, linking the external surface to the internal chorionic matrix and reinforcing the functional integration between structure and respiration. Despite this broadly conserved organization, significant interspecific variation is evident in several aspects of chorion architecture. These include differences in the relative thickness and degree of compaction of the C3/exochorion, the organization and density of the trabecular C1–C2 regions, and the arrangement and connectivity of aeropylar systems. For example, in *B. mori*, the chorion exhibits a highly ordered helicoidal lamellar structure with extensive protein crosslinking, resulting in a particularly robust and integrated architecture. In contrast, in species such as *U. ornatrix*, the transition between endochorion and exochorion is more gradual, forming a structural gradient rather than sharply defined layers. Similarly, comparative analyses of stored-product moths and noctuid pests have revealed variability in layer thickness and internal organization, which may influence permeability and physiological responses to environmental stressors [192,195,221,222,223].

In Hemiptera, the layered architecture of the chorion exhibits substantial diversity, combining a conserved multilayered framework with pronounced lineage-specific specializations. In Triatominae, particularly in *R. prolixus*, the chorion represents one of the most structurally complex examples described to date. Classical ultrastructural analyses reveal a highly stratified organization, comprising a vitelline membrane underlying a multilayered endochorion subdivided into several chemically and structurally distinct sublayers, including the inner polyphenol layer, the resistant protein layer, the outer polyphenol layer, the amber layer, and the soft protein layer, collectively conferring mechanical strength and controlled permeability. External to this, the exochorion consists of two lipoprotein layers, the soft and resistant exochorion layers, associated with polygonal surface patterning and traversed by pore canals that do not fully penetrate the inner layers. Notably, all seven layers are present throughout the egg, including both the mainbody and the operculum [19]. More recent analyses, however, describe a comparatively simplified organization, in which the chorion can be resolved into a thick outer layer with uniform electron density and a thinner, more porous inner layer, separated from a porous vitelline membrane by a subchorionic space [201], highlighting how methodological approaches and developmental stages can influence structural interpretation. Beyond *Rhodnius*, other hemipterans exhibit both shared and divergent architectural features. In *C. lectularius*, the chorion also comprises a vitelline membrane, followed by an innermost layer of porous struts, a middle layer with low electron density, and an outer electron-dense layer containing rounded electron-opaque regions near the surface. These layers show relatively little variation in thickness across the eggshell [203]. In the tarnished plant bug *Lygus lineolaris*, the eggshell is composed of a vitelline membrane, the chorion proper, and an innermost chorionic layer. The chorion proper includes an air layer subdivided into three distinct zones (I–III), exhibiting a columnar (colonnade-like) organization in which pillar-like elements delimit internal spaces, suggesting a structurally integrated system for gas diffusion [224]. Even more specialized configurations are observed in aquatic or semi-aquatic taxa such as the water scorpion *Nepa cinerea*, where the chorion incorporates a well-developed intrachorionic air space and external respiratory horns connected to an internal reticulated network of aeropylar channels, forming a plastron-like system that enables respiration under submerged conditions. This organization co-occurs with a typical endochorion associated with the vitelline envelope and an exochorion bearing tuberculate surface structures [225].

Within Orthoptera, the internal organization of the chorion reveals a spectrum of structural configurations, ranging from highly organized trabecular systems to more homogeneous fibrillar matrices, while still retaining a broadly comparable layered framework. In acridid species such as *L. migratoria*, the eggshell comprises a vitelline membrane and a chorion organized into two main regions: the endochorion and the exochorion. The endochorion is further subdivided into two regions: an actual endochorion, which appears to consist of a three-dimensional lattice of more or less tightly packed ribbon-like strips, often arranged in pairs, and an outer endochorion. These endochorionic strips are associated with a porous web-like assemblage of granules and clod-like structures of varying size, embedded in an electron-lucent matrix or interconnected by electron-dense, irregularly shaped, amorphous beams. The outer endochorion is more finely textured and may contain delicate rod-shaped double structures embedded within a fine granular matrix. The exochorion, in turn, consists of finely textured fibrous material, locally exhibiting disc-like formations or prominent tubercles [226]. In *S. gregaria*, the eggshell is sequentially secreted by follicular cells and consists of a vitelline membrane, an endochorion, and an exochorion. The endochorion is composed of crystalline tubules embedded in a granular matrix, whereas the exochorion forms a thick, fibrillar outer layer. Following oviposition, additional modifications occur in both the vitelline membrane and the endochorion, and an extrachorionic layer composed of curly fibers is deposited externally [52]. In *G. punctifrons*, the eggshell includes a vitelline membrane, an interchorionic layer, an air layer, and an outer chorionic layer. The outer chorionic layer contains thread-like proteinaceous structures interspersed with polysaccharide droplets, indicating a composite fibrillar organization [164]. In *E. plorans*, the chorion can be distinguished into a thin, relatively homogeneous exochorion containing numerous microporosities and a thicker endochorion exhibiting a complex and heterogeneous internal organization. The endochorion is structurally differentiated and comprises four main components: plates, granules, fibrils, and band-like elements, the latter displaying a crystalline arrangement. This layer is further subdivided into three distinct zones. The outermost zone, located immediately beneath the exochorion, is predominantly composed of plate-like structures. The intermediate zone contains band-like elements arranged in a dense network interspersed with plate-like structures and granular material. The innermost zone consists of one or a few band-like elements that progressively narrow into thin fibrils, between which fine granular material is distributed. Additionally, a waxy layer is present between the endochorion and the vitelline envelope [174].

Relevant studies investigating the cross-sectional organization of the chorion across insect taxa are summarized in Table S3.

## 6. Functional Implications and Applied Perspectives of Chorion Biogenesis

### 6.1. Functional Insights from Genetic and Molecular Disruption of Chorion Formation

Advances in molecular and genetic approaches have provided critical insights into the functional significance of chorion biogenesis, particularly through gene silencing/knockdown and loss-of-function studies. These investigations demonstrate that the integrity and functionality of the insect eggshell rely on a tightly coordinated network of structural proteins, enzymatic processes, and regulatory factors. Disruption of genes encoding vitelline membrane components, for instance, frequently compromises the initial scaffold of the eggshell, leading to defects in permeability barriers, impaired embryonic protection, and, in many cases, collapsed or/and malformed eggs with reduced viability. Such phenotypes have been reported in species including *D. melanogaster* [80,100] and the diamondback moth *Plutella xylostella* [227]. The functional importance of specific chorion proteins has also been extensively characterized. Structural components such as s36 [228], s38 [229], and the dec-1 gene [230] in *D. melanogaster*, as well as chorion proteins such as chorion protein (NlChP) in the brown planthopper *Nilaparvata lugens* [231], are essential for the proper assembly and spatial organization of chorionic layers. Disruption of these elements results in severe abnormalities in eggshell architecture, including defects in endochorion formation and exochorion patterning, often accompanied by reduced fertility.

Similarly, genes associated with pigmentation pathways, particularly members of the yellow gene family, play crucial roles in chorion melanization. Knockdown studies have revealed that impairment of these genes leads to abnormal chorion morphology and ultrastructure, increased susceptibility to desiccation, and decreased egg viability in diverse taxa, including the mosquito *Aedes albopictus* [232], the beetle *T. castaneum* [233], and *D. melanogaster* [86]. In parallel, enzymes involved in oxidative processes, such as peroxidases and laccases, are fundamental for stabilizing the chorion matrix through protein crosslinking reactions. Disruption of these enzymatic pathways often results in mechanically fragile or abnormally permeable eggshells [132,146,151,155], further emphasizing the importance of post-translational modifications in chorion maturation. Taken together, these findings demonstrate that chorion architecture is not merely a morphological outcome, but rather the direct manifestation of finely tuned molecular mechanisms. Alterations in specific proteins or enzymatic pathways are consistently translated into detectable structural changes at the ultrastructural level and profound consequences to embryonic development, reinforcing the concept that eggshell organization is intrinsically linked to its underlying molecular blueprint, underscoring the functional sensitivity of this system.

### 6.2. Biotechnological Potential and Implications for Vector and Pest Control

The functional vulnerability of chorion biogenesis pathways positions the insect eggshell as a promising target for biotechnological applications, particularly in the context of pest and vector control. Because successful embryogenesis critically depends on the correct formation of the chorion, disruption of key genes or biochemical pathways involved in its assembly can lead to complete reproductive failure, making chorion-associated molecules attractive candidates for targeted control strategies. Importantly, accumulating evidence indicates that many chorion proteins exhibit a high degree of species specificity, often showing limited homology to their counterparts in other taxa. This molecular divergence significantly enhances their potential as selective targets, as proteins that are broadly conserved across organisms are less suitable due to the risk of deleterious effects on non-target species, including vertebrates, pollinators, and beneficial predators [234,235]. Emerging approaches, including RNAi and gene-editing technologies, offer the potential to selectively disrupt genes involved in chorion formation in pest or vector species [236,237,238,239,240]. Targeting structural proteins and enzymes responsible for chorion hardening, or regulators of follicle cell differentiation, could impair eggshell integrity without directly affecting non-target organisms. Moreover, the relative accessibility of the eggshell as an external structure further supports its suitability as an intervention target. Consequently, proteins involved in chorion formation constitute attractive molecular targets for the development of species-specific control strategies based on gene-silencing technologies such as RNAi, as the examples discussed in the previous subsection. Despite these promising prospects, the application of chorion-targeted strategies remains largely underexplored, highlighting a significant opportunity for future translational research.

## 7. Final Remarks

In summary, this review provides an integrated view of insect chorion biogenesis, combining cellular, molecular, biochemical, and structural perspectives. Across taxa, choriogenesis emerges as a highly coordinated process involving follicle cell activity, regulated gene expression, and the sequential assembly of macromolecules, generating diverse chorion architectures while maintaining a conserved functional status. Importantly, the comparative approach adopted here highlights that, while a common architectural framework underlies chorion formation, extensive diversification occurs at multiple levels, from gene repertoires to ultrastructural organization, even among closely related species. This structural and molecular plasticity underscores the relevance of the chorion as a model system for studying evolutionary innovation, particularly in relation to reproductive strategies, environmental adaptation, and species diversification.

Beyond its fundamental biological significance, chorion biogenesis also holds considerable potential for applied research. The identification of key molecular determinants of eggshell formation, many of which are species-specific and essential for viability, provides promising avenues for the development of targeted strategies for vector and pest control. In this context, advances in functional genomics and gene-silencing technologies offer powerful tools to disrupt chorion formation in a selective and environmentally safe manner. However, many important knowledge gaps remain, particularly regarding the comprehensive molecular composition of the chorion and its integration across different levels of organization. Future studies combining high-resolution imaging with multi-omics approaches will be essential to fully elucidate the complexity of chorion assembly and function.

## Data Availability

Not applicable.

## Supplementary Materials

The following supporting information can be downloaded at https://www.mdpi.com/article/10.3390/insects17070659/s1. Table S1: Representative studies on the ultrastructural organization of follicle cells across insect taxa.; Table S2: Representative studies of external morphology of the chorion across insect taxa.; Table S3: Representative studies on cross-sectional organization of the chorion in different insect taxa.
