# Supplementary material for: From Follicle Cell Differentiation and Structure to Chorion Biogenesis in Insects: Cellular Mechanisms, Gene Regulation, Biochemical Composition and Structural Diversity
Source: Insects. 2026 Jun 23;17(7):659. doi: 10.3390/insects17070659 (PMC13409908; doi:10.3390/insects17070659)
Supplement: Supplementary file 1 [file insects-17-00659-s001.zip › Table S1-layout.pdf]

insects

MDPI

Supplementary Materials

From Follicle Cell Differentiation and Structure to Chorion Biogenesis in Insects: Cellular Mechanisms, Gene Regulation, Biochemical Composition and Structural Diversity

Table S1. Representative studies on the ultrastructural organization of follicle cells across insect taxa.

Order	Species	Reference
**Diptera**	*Drosophila melanogaster*	[1,2]
	Drosophila virilis	[3]
	Aedes aegypti	[4–6]
	Culex quinquefasciatus	[7]
	Ceratitis capitata	[8]
**Lepidoptera**	*Bombyx mori*	[9]
	Ephestia kühniella	[10,11]
	Pieris napi	[12]
	Diatraea saccharalis	[13]
	Galleria mellonella	[14]
**Hemiptera**	*Rhodnius prolixus*	[15,16]
	Mahanarva fimbriolata	[17]
	Lygus lineloaris	[18]
**Orthoptera**	*Schistocerca gregaria*	[19]
	Acheta domesticus	[20]
	Locusta migratória	[21]
	Gryllus yemma	[22]
**Coleoptera**	*Leptinotarsa decemlineata*	[23]
	Foucartia squamulata	[24]
**Odonata**	*Libellula depressa*	[25]
	Sympetrum frequens	[22]
**Hymenoptera**	*Eurytoma amygdali*	[26]
**Blattodea**	*Kalotermes flavicollis*	[27]
**Siphonaptera**	*Ctenocephalides felis*	[28]
**Plecoptera**	*Brachyptera risi*	[29]

1. Giorgi, F. An EM Autoradiographic Study on Ovarian Follicle Cells of Drosophila Melanogaster with Special Reference to the Formation of Egg Coverings. *Histochemistry* **1977**, *52*, 105–117, doi:https://doi.org/10.1007/BF00492287.
2. Papassideri, I.S.; Margaritis, L.H.; Gulik-Krzywicki, T. The Eggshell of Drosophila Melanogaster. VIII. Morphogenesis of the Wax Layer during Oogenesis. *TISSUE AND CELL.* **1993**, *25*, 929–936, doi:https://doi.org/10.1016/0040-8166(93)90041-I.
3. Trougakos, I.P.; Margaritis, L.H. The Formation of the Functional Chorion Structure of Drosophila Virilis Involves Intercalation of the "Middle" and "Late" Major Chorion Proteins into a Scaffold Formed by the "Early" Chorion Proteins: A General Model for Chorion Assembly in Drosophilidae. *J. Struct. Biol.* **1998**, *123*, 97–110, doi:https://doi.org/10.1006/jsbi.1998.4999.
4. Mathew, G.; Rat, K.S. Structure and Formation of Egg Membranes in Aedes Aegypti. (L.) (Diptera: Culicidae). *Int. J. Insect Morphol. Embryol.* **1975**, *4*, 369–380, doi:https://doi.org/10.1016/0020-7322(75)90037-9.
5. Raikhel, A.S.; Lea, A.O. Abnormal Vitelline Envelope Induced by Unphysiological Doses of Ecdysterone in Aedes Aegypti. *Physiol. Entomol.* **1982**, *7*, 55–64, doi:10.1111/j.1365-3032.1982.tb00666.x.
6. Raikhel, A.S.; Lea, A.O. Control of Follicular Epithelium Development and Vitelline Envelope Formation in the Mosquito; Role of Juvenile Hormone and 20-Hydroxyecdysone. *Tissue Cell* **1991**, *23*, 577–591, doi:https://doi.org/10.1016/0040-8166(91)90015-L.

7. Cardoso, A.F.; Cres, R.L.; Moura, A.S.; Almeida, F. de; Bijovsky, A.T. Culex Quinquefasciatus Vitellogenesis: Morphological and Biochemical Aspects. *Mem. Inst. Oswaldo Cruz* **2010**, *105*, 254–262, doi:https://doi.org/10.1590/S0074-02762010000300003.
8. Mouzaki, D.G.; Margaritis, L.H. Choriogenesis in the Medfly Ceratitis Capitata (Wiedermann) (Diptera: Tephritidae). *Int. J. Insect Morphol. Embryol.* **1991**, *20*, 51–68, doi:https://doi.org/10.1016/0020-7322(91)90027-7.
9. Yamauchi, H.; Yoshitake, N. Developmental Stages of Ovarian Follicles of the Silkworm, Bombyx Mori L. *J. Morphol.* **1984**, *179*, 21–31, doi:10.1002/jmor.1051790104.
10. Cruickshank, W.J. Ultrastructural Modifications in the Follicle Cells and Egg Membranes during Development of Flour Moth Oocytes. *J. Insect Physiol.* **1972**, *18*, 485–498, doi:https://doi.org/10.1016/0022-1910(72)90079-0.
11. Cummings, M.R. Formation of the Vitelline Membrane and Chorion in Developing Oocytes of Ephestia Kiihniella. *Zeitschrift für Zellforschung und Mikroskopische Anatomie* **1972**, *127*, 175–188.
12. Mazurkiewicz-Kania, M.; Simiczyjew, B.; Jędrzejowska, I. Differentiation of Follicular Epithelium in Polytrophic Ovaries of Pieris Napi (Lepidoptera: Pieridae)—How Far to Drosophila Model. *Protoplasma* **2019**, *256*, 1433–1447, doi:10.1007/s00709-019-01391-1.
13. dos Santos, D.C.; Gregorio, E.A. Deposition of the Eggshell Layers in the Sugar Cane Borer (Lepidoptera: Pyralidae): Ultrastructural Aspects. *Acta Microscopica* **2003**, *12*, 37–41.
14. Barbier, R.; Chauvin, G. Déterminisme de La Transformation de l◆enveloppe Vitelline Des Oeufs de Lépidoptères. *Int. J. Insect Morphol. Embryol.* **1977**, *6*, 171–178, doi:10.1016/0020-7322(77)90005-8.
15. Huebner, E.; Anderson, E. A Cytological Study of the Ovary of Rhodnius Prolixus. I. The Ontogeny of the Follicular Epithelium. *J. MORP* **1972**, *136*, 459–494, doi:https://doi.org/10.1002/jmor.1051360405.
16. Rios, T.; Bomfim, L.; Ramos, I. The Transition from Vitellogenesis to Choriogenesis Triggers the Downregulation of the UPR Sensors IRE1 and PERK and Alterations in the ER Architecture in the Follicle Cells of the Vector Rhodnius Prolixus. *Cell Tissue Res.* **2022**, *387*, 63–74, doi:10.1007/s00441-021-03547-z.
17. Caperucci, D.; Camargo-Mathias, M.I. Ultrastructural Study of the Ovary of the Sugarcane Spittlebug Mahanarva Fimbriolata (Hemiptera). *Micron* **2006**, *37*, 633–639, doi:https://doi.org/10.1016/j.micron.2006.02.002.
18. Ma, P.W.K.; Baird, S.; Ramaswamy, S.B. Morphology and Formation of the Eggshell in the Tarnished Plant Bug, Lygus Lineloaris (Palisot de Beauvois) (Hemiptera: Miridae). *Arthropod Struct. Dev.* **2002**, *31*, 131–146, doi:https://doi.org/10.1016/S1467-8039(02)00019-1.
19. Kimber, S.J. The Secretion of the Eggshell of Schistocerca Gregaria: Ultrastructure of the Follicle Cells during the Termination of Vitellogenesis and Eggshell Secretion. *J. Cell Sci* **1980**, *46*, 455–477, doi:https://doi.org/10.1242/jcs.46.1.455.
20. Dennis, J.C.; Bradley, J.T. Ovarian Follicle Development during Vitellogenesis in the House Cricket Acheta Domesticus. *J. Morphol.* **1989**, *200*, 185–198, doi:https://doi.org/10.1002/jmor.1052000208.
21. Bassemir, U. Ultrastructural Differentiations in the Developing Follicle Cortex of Locusta Migratoria, with Special Reference to Vitelline Membrane Formation. *Cell Tiss. Res* **1977**, *185*, 247–262, doi:https://doi.org/10.1007/BF00220669.
22. Matsuzaki, M. ELECTRON MICROSCOPIC STUDIES ON THE OOGENESIS OF DRAGONFLY AND CRICKET WITH SPECIAL REFERENCE TO THE PANOISTIC OVARIES. *Dev. Growth Differ.* **1971**, *13*, 379–398, doi:https://doi.org/10.1111/j.1440-169X.1971.00379.x.
23. De Loof, A.; Lagasse, A. The Ultrastructure of the Follicle Cells of the Ovary of the Colorado Beetle in Relation to Yolk Formation. *J. Insect Physiol.* **1970**, *16*, 211–220, doi:https://doi.org/10.1016/0022-1910(70)90162-9.
24. Bilifiski, S.; Petryszak, B. Cell and Tissue Research The Ultrastructure and Function of Follicle Cells in Foucartia Squamulata (Herbst) (Curculionidae). *Cell and Tissue Research* **1978**, *189*, 347–353, doi:https://doi.org/10.1007/BF00209282.
25. Gaino, E.; Piersanti, S.; Rebora, M. Egg Envelope Synthesis and Chorion Modification after Oviposition in the Dragonfly Libellula Depressa (Odonata, Libellulidae). *Tissue Cell* **2008**, *40*, 317–324, doi:https://doi.org/10.1016/j.tice.2008.02.005.
26. Mouzaki, D.G.; Margaritis, L.H. The Eggshell of the Almond Wasp Eurytoma Amygdali (Hymenoptera, Eurytomidae) - 1. Morphogenesis and Fine Structure of the Eggshell Layers. *Tissue Cell* **1994**, *26*, 559–568, doi:https://doi.org/10.1016/0040-8166(94)90008-6.
27. Grandi, G. Oogenesis in Kalotermes Flavicollis (Fabr.) (Isoptera, Kalotermitidae) III. Choriogenesis and Corpus Luteum Formation in Female Supplementary Reproductives. *Bolletino di zoologia* **1990**, *57*, 97–107, doi:https://doi.org/10.1080/11250009009355683.
28. Marchiondo, A.A.; Meola, S.M.; Palma, K.G.; Slusser, J.H.; Meola, R.W. Chorion Formation and Ultrastructure of the Egg of the Cat Flea (Siphonaptera: Pulicidae). *J. Med. Entomol.* **1999**, *36*, 149–157, doi:https://doi.org/10.1093/jmedent/36.2.149.

29. Michalik, A.; Rościszewska, E.; Miliša, M. The Structure and Ultrastructure of the Egg Capsule of Brachyptera Risi (Plecoptera, Nemouroidea, Taeniopterygidae) with Some Remarks Concerning Choriogenesis. *Microsc. Res. Tech.* **2015**, *78*, 180–186, doi:https://doi.org/10.1002/jemt.22459.
