# Supplementary material for: From Follicle Cell Differentiation and Structure to Chorion Biogenesis in Insects: Cellular Mechanisms, Gene Regulation, Biochemical Composition and Structural Diversity
Source: Insects. 2026 Jun 23;17(7):659. doi: 10.3390/insects17070659 (PMC13409908; doi:10.3390/insects17070659)
Supplement: Supplementary file 1 [file insects-17-00659-s001.zip › Table S2-layout.pdf]

insects

MDPI

Supplementary Materials

From Follicle Cell Differentiation and Structure to Chorion Biogenesis in Insects: Cellular Mechanisms, Gene Regulation, Biochemical Composition and Structural Diversity

Table S2. Representative studies of external morphology of the chorion across insect taxa.

Order	Species	Reference
Diptera	*Drosophila melanogaster*	[1,2]
	Drosophila species	[2–4]
	Aedes species	[5–9]
	Anopheles species	[10]
	Culex (Culex) saltanensis	[11]
	Sandflies species	[12,13]
	Psorophora albipes	[14]
	Psorophora cyanescens	[15]
	Haemagogus eucocelaenus	[16]
	Choerades species	[17]
	Laphria aurea	[17]
	Andrenosoma serratum	[17]
	Pollenia species	[18]
Lepidoptera	*Bombyx* species	[19]
	Manduca sexta	[20]
	Ephestia kühniella	[21,22]
	Cydia pomonella	[23]
	Heliothis virescens	[22,23]
	Spodoptera species	[22–24]
	Anticarsia gemmatalis	[22,24]
	Chrysodeixis includens	[24]
	Rachiplusia nu	[24]
	Helicoverpa species	[24]
	Chloridea virescens	[24]
	Euproctis chrysorrhoea	[25]
	Ariadne merione	[26]
	Nymphula nymphaeata	[27]
	Plodia interpunctella	[28]
	Sitotroga cerealella	[22]
	Corcyra cephalonica	[22]
	Diatraea saccharalis	[22]
	Samia ricini	[29]
Hemiptera	*Rhodnius* species	[30,31]
	Triatoma species	[32]
	Belminus species	[33]
	Cimex lectularius	[34]
	Ornithocoris pallidus	[35]
	Nezara viridula	[36]
	Abedus herberti	[37]
	Lycorma delicatula	[38]

**Orthoptera**	*Locusta migratoria*	[39]
	Gesonula punctifrons	[40]
	Boopedon diabolicum	[41]
	Schistocerca cohni	[41]
	Sphenarium purpurascens	[41]
	Brachystola mexicana	[41]
	Melanoplus differentialis	[41]
	Syrbula admirabilis	[41]
	Melanoplus lakinus	[41]
	Isophya nervosa	[42]
	Troglophilus species	[43]
	Tacbycines asynamorus	[43]
	Dolichopoda species	[43]
**Coleoptera**	*Tribolium castaneum*	[28]
	Diabrotica species	[44]
	Morimus funereus	[45]
**Odonata**	*Ictinogomphus rapax*	[46]
	Zyxomma petiolatum	[47]
	Aeshna juncea	[48]
	Tholymis tillarga	[49]
	Micrathyria hesperis	[50]
	Miathyria simplex	[50]
**Hymenoptera**	*Ampulex compressa*	[51]
	Hesperapis rhodocerata	[52]
**Siphonaptera**	*Ctenocephalides felis*	[53]
	Orchopeas leucopus	[53]
	Aetheca wagneri	[53]
	Conorhinopsylla stanfordi	[53]
	Epitedia faceta	[53]
	Sternopsylla distincta texana	[53]
	Craneopsylla minerva	[53]
**Plecoptera**	*Stenoperla prasina*	[54]
	Austroperla cyrene	[54]
	Zelandobius truncus	[54]
	Megaleptoperla grandis	[54]
	Acroperla trivacuata	[54]
	Isoperla species	[55]
**Ephemeroptera**	*Hermanella* species	[56]
	Traverella species	[56]
	Needhamella ehrhardt	[56]
	Hydrosmilodon saltensis	[56]
	Nousia delicata	[56]
	Ulmeritus carbonelli	[56]
	Hagenulus caligatus	[56]

1. Margaritis, L.H.; Kafatos, F.C.; Petrij, W.H. The Eggshell of Drosophila Melanogaster: I. Fine Structure of the Layers and Regions of the Wild-Type Eggshell. *J. Cell Sci* **1980**, *43*, 1–35, doi:https://doi.org/10.1242/jcs.43.1.1.
2. Jagadeeshan, S.; Singh, R.S. Rapid Evolution of Outer Egg Membrane Proteins in the Drosophila Melanogaster Subgroup: A Case of Ecologically Driven Evolution of Female Reproductive Traits. *Mol. Biol. Evol.* **2007**, *24*, 929–938, doi:10.1093/molbev/msm009.
3. Kambysellis, M.P. ULTRASTRUCTURE OF THE CHORION IN VERY CLOSELY RELATED DROSOPHILA SPECIES ENDEMIC TO HAWAII. *Syst. Biol.* **1974**, *23*, 507–512, doi:https://doi.org/10.1093/sysbio/23.4.507.

4. Kambysellis, M.P. Ultrastructural Diversity in the Egg Chorion of Hawaiian Drosophila and Scaptomyza: Ecological and Phylogenetic Considerations. *Int. J. Insect Morphol. Embryol.* **1993**, *22*, 417–446, doi:https://doi.org/10.1016/0020-7322(93)90022-S.
5. Bova, J.; Paulson, S.; Paulson, G. Morphological Differentiation of the Eggs of North American Container-Inhabiting Aedes Mosquitoes. *J. Am. Mosq. Control Assoc.* **2016**, *32*, 244–246, doi:https://doi.org/10.2987/15-6535.1.
6. Dos Santos-Mallet, J.R.; Müller, G.A.; Gleiser, R.M.; Alencar, J.; De Almeida Marques, W.; Sarmento, J.S.; Marcondes, C.B. Scanning Electron Microscopy of the Eggs of Aedes Scapularis from Southern South America. *J. Am. Mosq. Control Assoc.* **2010**, *26*, 205–209, doi:https://doi.org/10.2987/09-5959.1.
7. Linley, J.R. Comparative Fine Structure of the Eggs of Aedes Albopictus, Ae. Aegypti, and Ae. Bahamensis (Diptera: Culicidae). *J. Med. Entomol.* **1989**, *26*, 510–521, doi:https://doi.org/10.1093/jmedent/26.6.510.
8. Linley, J.R.; Clark, G.G. Egg of Aedes (Gymnometopa) Mediovittatus (Diptera: Culicidae). *J. Med. Entomol.* **1989**, *26*, 252–255, doi:https://doi.org/10.1093/jmedent/26.4.252.
9. Matsuo, K.; Yoshida, Y.; Lien, J.C. Scanning Electron Microscopy of Mosquitoes: II. The Egg Surface Structure of 13 Species of Aedes from Taiwan. *J. Med. Entomol.* **1974**, *11*, 179–188, doi:https://doi.org/10.1093/jmedent/11.2.179.
10. Valle, D.; Monnerat, A.T.; Soares, M.J.; Rosa-Freitas, M.G.; Pelajo-Machado, M.; Vale, B.S.; Lenzi, H.L.; Galler, R.; Lima, J.B.P. Mosquito Embryos and Eggs: Polarity and Terminology of Chorionic Layers. *J. Insect Physiol.* **1999**, *45*, 701–708, doi:https://doi.org/10.1016/S0022-1910(98)00154-1.
11. Santos-Mallet, J.R.; Balthazar, T.D.; Oliveira, A.A.; Marques, W.A.; Bastos, A.Q.; Freitas, S.P.C. The External Morphology of the Eggs of Culex (Culex) Saltanensis (Diptera: Culicidae) under Scanning Electron Microscopy. *J. Med. Entomol.* **2021**, *58*, 1134–1137, doi:https://doi.org/10.1093/jme/tjaa271.
12. Alencar, R.B.; Scarpassa, V.M. Morphology of the Eggs Surface of Ten Brazilian Species of Phlebotomine Sandflies (Diptera: Psychodidae). *Acta Trop.* **2018**, *187*, 182–189, doi:https://doi.org/10.1016/j.actatropica.2018.07.023.
13. Ward, R.D.; Ready, P.A. Chorionic Sculpturing in Some Sandfly Eggs (Diptera, Psychodidae). *Journal of Entomology Series A, General Entomology* **1975**, *50*, 127–134, doi:https://doi.org/10.1111/j.1365-3032.1975.tb00101.x.
14. Mello, C.F. De; Santos-Mallet, J.R.; Gleiser, R.M.; Alencar, J. Ultrastructure and Morphometry of the Egg of Psorophora Albipes (Theobald, 1907) (Diptera: Culicidae). *Zootaxa* **2017**, *4317*, 196–200, doi:https://doi.org/10.11646/zootaxa.4317.1.13.
15. García-Rejón, J.E.; Tzuc-Dzul, J.C.; Cigarroa-Toledo, N.; Talavera-Aguilar, L.G.; Cetina-Trejo, R.C.; Baak-Baak, C.M. Fecundity and Morphological Description of the Eggs of Psorophora Cyanescens (Coquillett, 1902) (Diptera: Culicidae) by Scanning Electron Microscopy. *Acta Zoológica Mexican* **2022**, *38*, 1–9, doi:10.21829/azm.2022.3812449.
16. Alencar, J.; Guimarães, A.É.; Mello, R.P.; Lopes, C.M.; Dégallier B E Jacenir, N.; Santos-Mallet, R. Scanning Electron Microscopy of Eggs of Haemagogus Leucocelaenus (Diptera: Culicidae). *Rev Saúde Pública* **2003**, *37*, 658–661, doi:10.1590/S0034-89102003000500017.
17. Hasbenlì, A.; Suludere, Z.; Candan, S.; Bayrakdar, F. Chorionic Structure of the Eggs of Five Laphriinae Species (Diptera: Asilidae) from Turkey. *J. Entomol. Res. Soc.* **2008**, *10*, 47–60.
18. Grzywacz, A.; Szpila, K.; Pape, T. Egg Morphology of Nine Species of Pollenia Robineau-Desvoidy, 1830 (Diptera: Calliphoridae). *Microsc. Res. Tech.* **2012**, *75*, 955–967, doi:https://doi.org/10.1002/jemt.22020.
19. Kawaguchi, Y.; Banno, Y.; Koga, K.; Kawarabata, T.; Doira, H. Comparison of Chorion Structure of Eggs from Bombyx Mori, Bombyx Mandarina (Lepidoptera:Bombycidae) and Their First FIlial Generation. *Appl. Entomol. Zool.* **1996**, *31*, 407–415, doi:10.1303/AEZ.31.407.
20. Orfanidou, C.C.; Hamodrakas, S.J.; Margaritis, L.H.; Galanopoulos, V.K.; Dedieu, J.C.; Gulik-Krzywicki, T. Fine Structure of the Chorion of Manduca Sexta and Sesamia Nonagrioides as Revealed by Scanning Electron Microscopy and Freeze-Fracturing. *Tissue Cell* **1992**, *24*, 735–744, doi:https://doi.org/10.1016/0040-8166(92)90045-9.
21. Cummings, M.R. Formation of the Vitelline Membrane and Chorion in Developing Oocytes of Ephestia Kiihniella. *Zeitschrift für Zellforschung und Mikroskopische Anatomie* **1972**, *127*, 175–188.
22. Cônsoli, F.L.; Kitajima, E.W.; Parra, J.R.P. Ultrastructure of the Natural and Factitious Host Eggs of Trichogramma Galloi Zucchi and Trichogramma Pretiosum Riley (Hymenoptera: Trichogrammatidae). *Int. J. Insect Morphol. Embryol.* **1999**, *28*, 211–231, doi:https://doi.org/10.1016/S0020-7322(99)00026-4.
23. Fehrenbacht, H.; Dittrich, V.; Zissler, D. Eggshell Fine Structure of Three Lepidopteran Pests: Cydia Pomonella (L.) (Tortricidae), Heliothis Virescens (Fabr.), and Spodoptera Littoralis (Boisd.) (Noctuidae). *Int. J. Insect Morphol. Embryol.* **1987**, *16*, 201–219, doi:https://doi.org/10.1016/0020-7322(87)90021-3.

24. Sosa-Gómez, D.R.; Specht, A.; Murúa, M.G.; de Jesus Andrade, C.G.T. External Microstructure of Eggs from Major Owlet Moth Pests (Lepidoptera: Noctuoidea) Associated with Brazilian Soybean Crops. *Rev. Bras. Entomol.* **2024**, *68*, doi:https://doi.org/10.1590/1806-9665-RBENT-2024-0079.
25. Candan, S.; Suludere, Z.; Bayrakdar, F. Surface Morphology of Eggs of Euproctis Chrysorrhoea (Linnaeus, 1758). *Acta Zoologica* **2008**, *89*, 133–136, doi:https://doi.org/10.1111/j.1463-6395.2007.00300.x.
26. Srivastava, A.K.; Kumar, K. Ultrastructure of Egg Chorion of Castor Butterfly Ariadne Merione (Crammer) (Lepidoptera: Nymphalidae). *Zoologischer Anzeiger - A Journal of Comparative Zoology* **2016**, *263*, 1–5, doi:https://doi.org/10.1016/j.jcz.2016.03.015.
27. Barbier, R.; Chauvin, G. The Aquatic Egg of Nymphula Nymphaeata (Lepidoptera: Pyralidae). *Cell and Tissue Research* **1974**, *149*, 473–479, doi:https://doi.org/10.1007/BF00223026.
28. Gautam, S.G.; Opit, G.P.; Margosan, D.; Hoffmann, D.; Tebbets, J.S.; Walse, S. Comparative Egg Morphology and Chorionic Ultrastructure of Key Stored-Product Insect Pests. *Ann. Entomol. Soc. Am.* **2014**, *108*, 43–56, doi:https://doi.org/10.1093/aesa/sau001.
29. Renthlei, C.Z.; Raghuvarman, A.; Kharbuli, B.; Dey, S. Progressive Chorion Morphology during Egg Development in Samia Ricini (Donovan). *Microsc. Res. Tech.* **2010**, *73*, 234–239, doi:https://doi.org/10.1002/jemt.20781.
30. Bomfim, L.; Vieira, P.; Fonseca, A.; Ramos, I. Eggshell Ultrastructure and Delivery of Pharmacological Inhibitors to the Early Embryo of R. Prolixus by Ethanol Permeabilization of the Extraembryonic Layers. *PLoS One* **2017**, *12*, e0185770, doi:10.1371/journal.pone.0185770.
31. dos Santos, C.M.; Jurberg, J.; Galvão, C.; da Rosa, J.A.; Júnior, W.C.; Barata, J.M.; Obara, M.T. Comparative Descriptions of Eggs from Three Species of Rhodnius (Hemiptera: Reduviidae: Triatominae). *Mem. Inst. Oswaldo Cruz* **2009**, *104*, 1012–1018, doi:https://doi.org/10.1590/S0074-02762009000700013.
32. Obara, M.T.; Da Rosa, J.A.; Da Silva, N.N.; Ceretti, W.; Urbinatti, P.R.; Barata, J.M.S.; Jurberg, J.; Galvão, E.C. Morphological and Histological Study of Eggs of Six Species of the Triatoma Genus (Hemiptera: Reduviidae). *Neotrop. Entomol.* **2007**, *36*, 798–806, doi:https://doi.org/10.1590/S1519-566X2007000500023.
33. Sandoval, C.M.; Nieves, E.; Angulo, V.M.; Aristeu, J.; Rosa, D.A.; Aldana, E. Morphology of the Eggs of the Genus Belminus (Hemiptera: Reduviidae: Triatominae) by Optical and Scanning Electron Microscopy. *Zootaxa* **2011**, *2970*, 33–40, doi:https://doi.org/10.11646/zootaxa.2970.1.2.
34. Baker, G.T.; Lawrence, A.; Kuklinski, R.; Goddard, J. Morphological and Ultrastructural Characteristics of the Chorion of Cimex Lectularius Linnaeus (Hemiptera: Cimicidae). *Proc. Entomol. Soc. Wash.* **2013**, *115*, 325–332, doi:https://doi.org/10.4289/0013-8797.115.4.325.
35. Bastos, A.Q.; Freitas, S.P.C.; Lopes, C.M.; Gonçalves, T.C.M.; Santos-Mallet, J.R. Morphology of the Eggs of Ornithocoris Pallidus (Hemiptera, Cimicidae, Haematosiphoninae). *J. Med. Entomol.* **2021**, *58*, 486–488, doi:https://doi.org/10.1093/jme/tjaa168.
36. Baral, A.; Piersanti, S.; Salerno, G.; Gorb, S.; Rebora, M. Egg Glue Secretion and Chorion Morphology of the Southern Green Stinkbug Nezara Viridula L. (Hemiptera: Pentatomidae). *Arthropod Struct. Dev.* **2025**, *89*, 101493, doi:https://doi.org/10.1016/j.asd.2025.101493.
37. Goforth, C.L.; Smith, R.L. Respiratory Morphology of the Abedus Herberti Hidalgo Egg Chorion (Hemiptera: Belostomatidae). *J. Morphol.* **2011**, *272*, 796–801, doi:https://doi.org/10.1002/jmor.10945.
38. Powell, J.M.; Nixon, L.J.; Lourie, A.P.; Leskey, T.C.; Walse, S.S. Egg Morphology and Chorionic Ultrastructure of Spotted Lanternfly, Lycorma Delicatula (White) (Hemiptera: Fulgoridae). *Forests* **2023**, *14*, 2354, doi:https://doi.org/10.3390/f14122354.
39. Roonwal, M.L. The Growth-Changes and Structure of the Egg of the African Migratory Locust, Locusta Migratoria Migratorioides, R. & F. (Orthoptera, Acrididae). *Bull. Entomol. Res.* **1936**, *27*, 1–14, doi:https://doi.org/10.1017/S0007485300058089.
40. Shyam Roy, A.; Ghosh, D. Ultrastructure, Solubilization and Protein Composition of Eggshell (Chorion) of Gesonula Punctifrons (Stal, 1861) (Orthoptera: Acrididae). *J. Entomol. Res. Soc.* **2014**, *16*, 45–54.
41. Salas-Araiza, M.D.; MacKay, W.P.; Valdez-Carrasco, J.; Salazar-Solís, E.; Martínez-Jaime, O.A. Characterization and Comparison of the Eggs of Seven Species of Mexican Grasshoppers. *Southwestern Entomologist* **2013**, *38*, 267–274, doi:https://doi.org/10.3958/059.038.0210.
42. Amutkan Mutlu, D.; Suludere, Z. Ultrastructural Changes in the Ovariole of Isophya Nervosa Ramme, 1931 (Orthoptera: Tettigoniidae) and Egg Morphology. *Microscopy and Microanalysis* **2022**, *28*, 837–843, doi:https://doi.org/10.1017/S1431927622000319.
43. Rampini, M.; Saltini, G. Observations on the Egg Ultrastructure of Some Rhaphidophoridae (Orthoptera) of the Mediterranean Area. *Bollettino Di Zoologia* **1994**, *61*, 1–8, doi:https://doi.org/10.1080/11250009409355853.

44. Rowley, W.A.; Peters, D.C. Scanning Electron Microscopy of the Eggshell of Four Species of Diabrotica (Coleoptera: Chrysomelidae). *Ann. Entomol. Soc. Am.* **1972**, *65*, 1188–1191, doi:https://doi.org/10.1093/aesa/65.5.1188.
45. Çiftçi, D. Ultrastructure of Egg Chorion of Vulnerable Species Morimus Funereus Mulsant, 1862 (Coleoptera: Cerambycidae). *Hacettepe Journal of Biology and Chemistry* **2018**, *46*, 315–319.
46. Andrew, R.J.; Tembhare, D.B. Surface Ultrastructure of the Egg Chorion in the Dragonfly, Ictinogomphus Rapax (Rambur) (Odonata: Gomphidae). *Int. J. Insect Morphol. Embryol.* **1992**, *21*, 347–350, doi:https://doi.org/10.1016/0020-7322(92)90029-M.
47. Andrew, R.J.; Tembharef, D.B. Ultrastructural Post-Oviposition Changes in the Egg Chorion of the Dragon-Fly, Zyxomma Petiolatum Rambur (Odonata: Libellulidae). *Int. J. Insect Morphol. Embryol.* **1995**, *24*, 235–238, doi:https://doi.org/10.1016/0020-7322(94)00019-M.
48. Sahlén, G. Ultrastructure of the Eggshell of Aeshna Juncea (L.) (Odonata : Aeshnidae). *Int. J. Insect Morphol. Embryol.* **1994**, *23*, 345–354, doi:https://doi.org/10.1016/0020-7322(94)90030-2.
49. Miller, P.L.; Miller, A.K. Rates of Oviposition and Some Other Aspects of Reproductive Behaviour in Tholymis Tillarga (Fabricius) in Kenya (Anisoptera: Libellulidae). *Odonatologica* **1985**, *14*, 287–299.
50. Giraldin, M.M.; Bernardy, J.V.; de Azevedo Brito, P.V.; De Marco Júnior, P. Egg Morphology of Two Neotropical Dragonflies: Micrathyria Hesperis and Miathyria Simplex (Odonata: Libellulidae). *Neotrop. Entomol.* **2023**, *52*, 1109–1118, doi:https://doi.org/10.1007/s13744-023-01081-x.
51. Gnatzy, W.; Volknandt, W.; Dzwoneck, A. Egg-Laying Behavior and Morphological and Chemical Characterization of Egg Surface and Egg Attachment Glue of the Digger Wasp Ampulex Compressa (Hymenoptera, Ampulicidae). *Arthropod Struct. Dev.* **2018**, *47*, 74–81, doi:https://doi.org/10.1016/j.asd.2017.11.010.
52. Rozen, J.G. Hesperapis Rhodocerata: Behavioral Biology, Egg, and Larval Instars, Including Behavioral and Larval Comparisons with H. Larreae (Hymenoptera: Melittidae: Dasypodainae). *Am. Mus. Novit.* **2016**, *2016*, 1–19, doi:https://doi.org/10.1206/3856.1.
53. Linley, J.R.; Benton, A.H.; Day, J.F. Ultrastructure of the Eggs of Seven Flea Species (Siphonaptera). *J. Med. Entomol.* **1994**, *31*, 813–827, doi:https://doi.org/10.1093/jmedent/31.6.813.
54. Mtow, S.; Smith, B.J.; Machida, R. Egg Structure of Five Antarctoperlarian Stoneflies (Insecta: Plecoptera, Antarctoperlaria). *Arthropod Struct. Dev.* **2021**, *60*, 101011, doi:https://doi.org/10.1016/j.asd.2020.101011.
55. Michalik, A.; Miliša, M.; Michalik, K.; Rościszewska, E. The Structure and Ultrastructure of the Egg Capsules of Stoneflies of the Genus Isoperla (Insecta, Plecoptera, Perlodidae). *Microsc. Res. Tech.* **2017**, *80*, 1234–1246, doi:https://doi.org/10.1002/jemt.22922.
56. Domínguez, E.; Cuezzo, M.G. Ephemeroptera Egg Chorion Characters: A Test of Their Importance in Assessing Phylogenetic Relationships. *J. Morphol.* **2002**, *253*, 148–165, doi:https://doi.org/10.1002/jmor.1117.
