# Supplementary material for: From Follicle Cell Differentiation and Structure to Chorion Biogenesis in Insects: Cellular Mechanisms, Gene Regulation, Biochemical Composition and Structural Diversity
Source: Insects. 2026 Jun 23;17(7):659. doi: 10.3390/insects17070659 (PMC13409908; doi:10.3390/insects17070659)
Supplement: Supplementary file 1 [file insects-17-00659-s001.zip › Table S3-layout.pdf]

insects

MDPI

Supplementary Materials

From Follicle Cell Differentiation and Structure to Chorion Biogenesis in Insects: Cellular Mechanisms, Gene Regulation, Biochemical Composition and Structural Diversity

Table S3. Representative studies on cross-sectional organization of the chorion in different insect taxa.

Order	Species	Reference
**Diptera**	*Drosophila melanogaster*	[1–3]
	Drosophila grimshawi	[4]
	Dacus oleae	[5]
	Ceratitis capitata	[6]
	Megaselia species	[7]
	Aedes aegypti	[8,9]
	Anopheles species	[9]
	Toxorhynchites splendens	[9]
	Culex species	[9,10]
**Lepidoptera**	*Bombyx mori*	[11]
	Sitotroga cerealella	[12]
	Ephestia kuehniella	[12]
	Corcyra cephalonica	[12]
	Diatraea saccharalis	[12]
	Heliothis virescens	[12,13]
	Anticarsia gemmatalis	[12]
	Spodoptera species	[12,13]
	Cydia pomonella	[13]
	Plodia interpunctella	[14]
	Amyelois transitella	[14]
	Utetheisa ornatrix	[15]
**Hemiptera**	*Rhodnius prolixus*	[16,17]
	Cimex lectularius	[18]
	Lygus lineolaris	[19]
	Nepa cinerea	[20]
**Orthoptera**	*Locusta migratoria*	[21]
	Schistocerca gregária	[22]
	Gesonula punctifrons	[23]
	Oxya hyla hyla	[24]
	Eyprepocnemis plorans	[25]
**Coleoptera**	*Tribolium castaneum*	[14]
	Carpophilus hemipterus	[14]
	Sitophilus granarius	[26]
	Listronotus oregonensis	[27]
**Odonata**	*Aeshna juncea*	[28]
	Zyxomma petiolatum	[29]
	Micrathyria hesperis	[30]
	Miathyria simplex	[30]
	Sympetrum frequens	[31]
	Libellula depressa	[32]

**Hymenoptera**	*Cardiochiles nigriceps*	[33]
	Meteorus pulchricornis	[34]
**Siphonaptera**	*Ctenocephalides felis*	[35]
**Plecoptera**	*Stenoperla prasina*	[36]
	Austroperla cyrene	[36]
	Zelandobius truncus	[36]
	Brachyptera risi	[37]
	Isoperla species	[38]

1. Hamodrakas, S.J.; Margaritis, L.H.; Nixon, P.E. Crystalline Layer in Drosophila Melanogaster Eggshell: Arrangement of Components as Revealed by Negative Staining and Reconstruction. *Int. J. Biol. Macromol.* **1982**, *4*, 25–31, doi:https://doi.org/10.1016/0141-8130(82)90006-X.
2. Margaritis, L.H. The Eggshell of Drosophila Melanogaster. II. New Staging Characteristics and Fine Structural Analysis of Choriogenesis. *Can. J. Zool.* **1986**, *64*, doi:https://doi.org/10.1139/z86-330.
3. Margaritis, L.H.; Kafatos, F.C.; Petrij, W.H. The Eggshell of Drosophila Melanogaster: I. Fine Structure of the Layers and Regions of the Wild-Type Eggshell. *J. Cell Sci* **1980**, *43*, 1–35, doi:https://doi.org/10.1242/jcs.43.1.1.
4. Margaritis, L.H.; Dellas, K.; Kalantzi, M.C.; Kambysellis, M.P. The Eggshell of Hawaiian Drosophila: Structural and Biochemical Studies in D. Grimshawi and Comparison to D. Melanogaster. *Wilhelm Roux's archives of developmental biology* **1983**, *192*, 303–316, doi:https://doi.org/10.1007/BF00848810.
5. Mouzaki, D.G.; Zarani, F.E.; Margaritis, L.H. Structure and Morphogenesis of the Eggshell and Micropylar Apparatus in the Olive Fly, Dacus Oleae (Diptera: Tephritidae). *J. Morphol.* **1991**, *209*, 39–52, doi:https://doi.org/10.1002/jmor.1052090105.
6. Mouzaki, D.G.; Margaritis, L.H. Choriogenesis in the Medfly Ceratitis Capitata (Wiedermann) (Diptera: Tephritidae). *Int. J. Insect Morphol. Embryol.* **1991**, *20*, 51–68, doi:https://doi.org/10.1016/0020-7322(91)90027-7.
7. Wolf, K.W.; Liu, G. Fine Structure of the Egg-Shell in Two Humpbacked Flies, Megaselia Scalaris and Megaselia Spiracularis (Diptera: Phoridae). *Int. J. Insect Morphol. Embryol.* **1996**, *25*, 289–294, doi:https://doi.org/10.1016/0020-7322(96)00001-3.
8. Mundim-Pombo, A.P.M.; Carvalho, H.J.C. de; Rodrigues Ribeiro, R.; León, M.; Maria, D.A.; Miglino, M.A. Aedes Aegypti: Egg Morphology and Embryonic Development. *Parasit. Vectors* **2021**, *14*, 531, doi:https://doi.org/10.1186/s13071-021-05024-6.
9. Sahlén, G. Eggshell Ultrastructure in Four Mosquito Genera (Diptera, Culicidae). *J. Am. Mosq. Control Assoc.* **1996**, *12*, 263–270.
10. Sahlén, G. Egg Raft Adhesion and Chorion Structure in Culex Pipiens L. (Diptera : Culicidae). *Int. J. Insect Morphol. Embryol.* **1990**, *19*, 307–314, doi:https://doi.org/10.1016/0020-7322(90)90015-H.
11. Papanikolaou, A.M.; Margaritis, L.H.; Hamodrakas, S.J. Ultrastructural Analysis of Chorion Formation in the Silkmoth Bombyx Mori. *Can. J. Zool.* **1986**, *64*, doi:https://doi.org/10.1139/z86-175.
12. Cônsoli, F.L.; Kitajima, E.W.; Parra, J.R.P. Ultrastructure of the Natural and Factitious Host Eggs of Trichogramma Galloi Zucchi and Trichogramma Pretiosum Riley (Hymenoptera: Trichogrammatidae). *Int. J. Insect Morphol. Embryol.* **1999**, *28*, 211–231, doi:https://doi.org/10.1016/S0020-7322(99)00026-4.
13. Fehrenbacht, H.; Dittrich, V.; Zissler, D. Eggshell Fine Structure of Three Lepidopteran Pests: Cydia Pomonella (L.) (Tortricidae), Heliothis Virescens (Fabr.), and Spodoptera Littoralis (Boisd.) (Noctuidae). *Int. J. Insect Morphol. Embryol.* **1987**, *16*, 201–219, doi:https://doi.org/10.1016/0020-7322(87)90021-3.
14. Gautam, S.G.; Opit, G.P.; Margosan, D.; Hoffmann, D.; Tebbets, J.S.; Walse, S. Comparative Egg Morphology and Chorionic Ultrastructure of Key Stored-Product Insect Pests. *Ann. Entomol. Soc. Am.* **2014**, *108*, 43–56, doi:https://doi.org/10.1093/aesa/sau001.
15. Wolf, K.W.; Murphy, C.; Reid, W.; Garraway, E. Fine Structure of the Eggshell in Utetheisa Ornatrix (Lepidoptera: Arctiidae). *Invertebr. Reprod. Dev.* **2000**, *38*, 85–94, doi:https://doi.org/10.1080/07924259.2000.9652442.
16. Beament, J.W.L. The Formation and Structure of the Chorion of the Egg in an Hemipteran, Rhodnius Prolixus. *J Cell Sci* **1946**, *348*, 393–439, doi:https://doi.org/10.1242/jcs.s2-87.348.393.
17. Bomfim, L.; Vieira, P.; Fonseca, A.; Ramos, I. Eggshell Ultrastructure and Delivery of Pharmacological Inhibitors to the Early Embryo of R. Prolixus by Ethanol Permeabilization of the Extraembryonic Layers. *PLoS One* **2017**, *12*, e0185770, doi:10.1371/journal.pone.0185770.
18. Baker, G.T.; Lawrence, A.; Kuklinski, R.; Goddard, J. Morphological and Ultrastructural Characteristics of the Chorion of Cimex Lectularius Linnaeus (Hemiptera: Cimicidae). *Proc. Entomol. Soc. Wash.* **2013**, *115*, 325–332, doi:https://doi.org/10.4289/0013-8797.115.4.325.

19. Ma, P.W.K.; Baird, S.; Ramaswamy, S.B. Morphology and Formation of the Eggshell in the Tarnished Plant Bug, Lygus Lineloaris (Palisot de Beauvois) (Hemiptera: Miridae). *Arthropod Struct. Dev.* **2002**, *31*, 131–146, doi:https://doi.org/10.1016/S1467-8039(02)00019-1.
20. Ozdamar, H.; Candan, S.; Özyurt Koçakoğlu, N.; Arslan, H. Functions and Morphological Structure of Respiratory Horns and Eggs of the Biocontrol Agent Nepa Cinerea Linnaeus, 1758 (Hemiptera: Nepidae). *Microscopy and Microanalysis* **2025**, *31*, ozaf061, doi:https://doi.org/10.1093/mam/ozaf061.
21. Rinterknecht, E. The Fine Structure of the Eggshell of the Laid Egg of Locusta Migratoria Migratorioides. *Tissue Cell* **1993**, *25*, 599–610, doi:https://doi.org/10.1016/0040-8166(93)90012-A.
22. Kimber, S.J. The Secretion of the Eggshell of Schistocerca Gregaria: Ultrastructure of the Follicle Cells during the Termination of Vitellogenesis and Eggshell Secretion. *J. Cell Sci* **1980**, *46*, 455–477, doi:https://doi.org/10.1242/jcs.46.1.455.
23. Shyam Roy, A.; Ghosh, D. Ultrastructure, Solubilization and Protein Composition of Eggshell (Chorion) of Gesonula Punctifrons (Stal, 1861) (Orthoptera: Acrididae). *J. Entomol. Res. Soc.* **2014**, *16*, 45–54.
24. Roy, A.S.; Ghosh, D. Chorion Is a Complex Structure of Protein and Polysaccharide-a Microscopical Study in Oxya Hyla Hyla (Orthoptera: Acrididae) (Serville, 1831). *J. Entomol. Zool. Stud.* **2014**, *2*, 144–150.
25. Viscuso, R.; Longo, G.; Giuffrida, A. Ultrastructural Features of Chorion and Micropyles in Eggs of Eyprepocnemis Plorans (Orthoptera, Acrididae). *Bolletino di zoologia* **1990**, *57*, 303–308, doi:https://doi.org/10.1080/11250009009355712.
26. Gaino, E.; Fava, A. Egg General Morphology and Eggshell Fine Organization of the Grain Weevil Sitophilus Granarius (L.) (Coleoptera: Curculionidae). *Entomologica* **1995**, *29*, 87–98, doi:https://doi.org/10.15162/0425-1016/658.
27. Nénon, J.P.; Boivin, G.; Allo, M.R. Fine Structure of the Egg Envelopes in Listronotus Oregonensis (Leconte) (Coleoptera: Curculionidae) and Morphological Adaptations to Oviposition Sites. *Int. J. Insect Morphol. Embryol.* **1995**, *24*, 333–342, doi:https://doi.org/10.1016/0020-7322(94)00023-J.
28. Sahlén, G. Ultrastructure of the Eggshell of Aeshna Juncea (L.) (Odonata : Aeshnidae). *Int. J. Insect Morphol. Embryol.* **1994**, *23*, 345–354, doi:https://doi.org/10.1016/0020-7322(94)90030-2.
29. Andrew, R.J.; Tembharef, D.B. Ultrastructural Post-Oviposition Changes in the Egg Chorion of the Dragon-Fly, Zyxomma Petiolatum Rambur (Odonata: Libellulidae). *Int. J. Insect Morphol. Embryol.* **1995**, *24*, 235–238, doi:https://doi.org/10.1016/0020-7322(94)00019-M.
30. Giraldin, M.M.; Bernardy, J.V.; de Azevedo Brito, P.V.; De Marco Júnior, P. Egg Morphology of Two Neotropical Dragonflies: Micrathyria Hesperis and Miathyria Simplex (Odonata: Libellulidae). *Neotrop. Entomol.* **2023**, *52*, 1109–1118, doi:https://doi.org/10.1007/s13744-023-01081-x.
31. Matsuzaki, M. ELECTRON MICROSCOPIC STUDIES ON THE OOGENESIS OF DRAGONFLY AND CRICKET WITH SPECIAL REFERENCE TO THE PANOISTIC OVARIES. *Dev. Growth Differ.* **1971**, *13*, 379–398, doi:https://doi.org/10.1111/j.1440-169X.1971.00379.x.
32. Gaino, E.; Piersanti, S.; Rebora, M. Egg Envelope Synthesis and Chorion Modification after Oviposition in the Dragonfly Libellula Depressa (Odonata, Libellulidae). *Tissue Cell* **2008**, *40*, 317–324, doi:https://doi.org/10.1016/j.tice.2008.02.005.
33. Davies, D.H.; Burghardt, R.L.; Vinson, S.B. Oogenesis of Cardiochiles Nigriceps Viereck (Hymenoptera : Braconidae): Histochemistry and Development of the Chorion with Special Reference to the Fibrous Layer. *Int. J. Insect Morphol. Embryol.* **1986**, *15*, 363–374, doi:https://doi.org/10.1016/0020-7322(86)90030-9.
34. Chen, Y.; Wang, P.; Shu, X.; Wang, Z.; Chen, X. Morphology and Ultrastructure of the Female Reproductive Apparatus of an Asexual Strain of the Endoparasitoid Meteorus Pulchricornis (Wesmael) (Hymenoptera, Braconidae). *Biology (Basel).* **2023**, *12*, 713, doi:https://doi.org/10.3390/biology12050713.
35. Marchiondo, A.A.; Meola, S.M.; Palma, K.G.; Slusser, J.H.; Meola, R.W. Chorion Formation and Ultrastructure of the Egg of the Cat Flea (Siphonaptera: Pulicidae). *J. Med. Entomol.* **1999**, *36*, 149–157, doi:https://doi.org/10.1093/jmedent/36.2.149.
36. Mtow, S.; Smith, B.J.; Machida, R. Egg Structure of Five Antarctoperlarian Stoneflies (Insecta: Plecoptera, Antarctoperlaria). *Arthropod Struct. Dev.* **2021**, *60*, 101011, doi:https://doi.org/10.1016/j.asd.2020.101011.
37. Michalik, A.; Rościszewska, E.; Miliša, M. The Structure and Ultrastructure of the Egg Capsule of Brachyptera Risi (Plecoptera, Nemouroidea, Taeniopterygidae) with Some Remarks Concerning Choriogenesis. *Microsc. Res. Tech.* **2015**, *78*, 180–186, doi:https://doi.org/10.1002/jemt.22459.
38. Michalik, A.; Miliša, M.; Michalik, K.; Rościszewska, E. The Structure and Ultrastructure of the Egg Capsules of Stoneflies of the Genus Isoperla (Insecta, Plecoptera, Perlodidae). *Microsc. Res. Tech.* **2017**, *80*, 1234–1246, doi:https://doi.org/10.1002/jemt.22922.
